# Ontogeny-specific induction of the *KMT2A::AFF1*-fusion drives development of a distinct CD24 positive pre-leukemic state

**DOI:** 10.1038/s41375-025-02665-9

**Published:** 2025-07-11

**Authors:** Ariana S. Calderón, Roshanak Ghazanfari, Zahra Masoumi, Shabnam Kharazi, Sara Palo, Stefan Lang, Kristijonas Žemaitis, Mohamed Eldeeb, Agatheeswaran Subramaniam, Shamit Soneji, Ronald W. Stam, David Bryder, Charlotta Böiers

**Affiliations:** 1 Division of Molecular Hematology, Lund Stem Cell Center, Lund University, Lund, Sweden; 2 Division of Molecular Medicine and Gene Therapy, Lund Stem Cell Center, Lund University, Lund, Sweden; 3 Princess Maxima Center for Pediatric Oncology, Utrecht, The Netherlands

**Keywords:** Cancer stem cells, Lymphopoiesis

## Abstract

Infant Acute Lymphoblastic Leukemia (ALL) driven by the *KMT2A::AFF1* onco-fusion is an aggressive, poor prognosis disease with few co-operative mutations. The fusion originates *in utero*, yet the embryonic initiating steps of disease development remain poorly understood. Here, we present a novel murine *KMT2A::AFF1* model, that provides key insights into *KMT2A::AFF1* pre-leukemia, relevant to human disease. The model enables precise oncogene induction, and upon targeting hematopoietic stem and progenitor cells (HSPCs) a selective negative impact on proliferation of hematopoietic stem cells (HSCs) was observed, regardless of developmental state during induction. However, a unique CD24^+^PreProB subset expanded exclusively within the *KMT2A::AFF1* embryonic context. This population was absent when targeting lymphoid progenitors, highlighting the importance of the cell of origin for leukemic development. The CD24^+^PreProB subset displayed key features of pre-leukemic stem cells, including lineage plasticity and aberrant engraftment ability. In line with their pre-malignant phenotype, single-cell transcriptomics revealed a signature consistent with stemness, and notable, up-regulation of *Hmga2*, a regulator of self-renewal. The signature was critically transferable to human KMT2A::AFF1 patients. Furthermore, given that CD24 is a potential therapeutic target, our findings uncover a distinct embryonic pre-leukemic state with direct relevance to human disease.

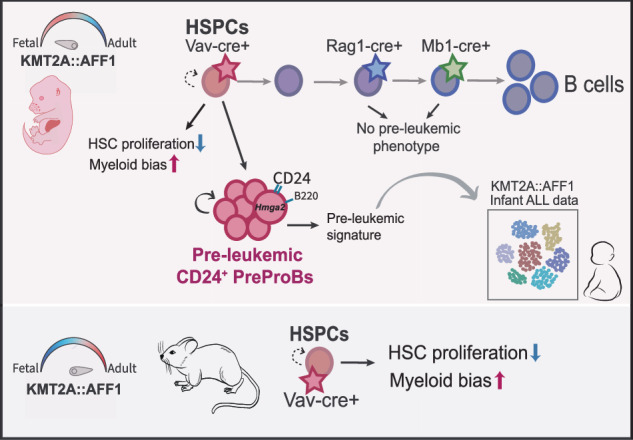

## Introduction

Despite a remarkable progress in treating children with leukemia, infants with acute lymphoblastic leukemia (ALL) still have a poor prognosis, with survival rates of about fifty percent [1, 2]. These infant leukemias are often driven by chromosomal translocations of the *KMT2A* gene with *AFF1 (t(4;11)*), and the resulting *KMT2A::AFF1* oncogene (*MLL::AF4)* gives rise to an aggressive, poor prognosis B cell leukemia [1–4]. *KMT2A* is a histone methyltransferase with key roles in epigenetic regulation, and the fusion drives transcriptional activation and aberrant expression of Homeobox *(HOX*) genes, contributing to leukemogenesis [5, 6]. *KMT2A::AFF1* leukemia is seen in all age groups, but the disease in infants can be regarded as its own entity, with a particularly low mutational burden, and a rapid and therapy resistant disease [3, 7].

There is abundant evidence that infant ALL originates prenatally [8, 9], and after a pre-leukemic phase, disease becomes evident before one year of age. Embryonic hematopoiesis occurs in overlapping waves, and in contrast to adult hematopoiesis, embryonic progenitors emerge independently of definitive hematopoietic stem cells (HSCs) [10]. Definitive HSCs are produced in the aorta–gonad–mesonephros (AGM) region at embryonic day (E) 10.5 in mice, and then migrate to the fetal liver (FL), which serves as the main hematopoietic organ for most of development, whereas the bone marrow (BM) becomes active primarily after birth [10].

Infant ALL originates within this embryonic context, and a relevant model of the disease onset requires that the initiating onco-fusion is expressed at the appropriate developmental stage and in the relevant cell type(s). Earlier studies have mainly focused on the adult setting [11–15] and when investigations on early embryonic hematopoietic cells were performed, these findings were limited by the fact that no phenotype was observed in the embryos [16]. The impact of the oncogene was detectable only ex vivo, and in adult mice, it manifested as a lymphoma-like condition rather than leukemia [16]. Recent advancements in transforming human cells have shown promise [17, 18], though these models also face limitations in exploring the pre-leukemic state of the disease. Consequently, as of yet, the prenatal initiating phase of infant ALL remains a challenge to model in situ, and the events preceding leukemia development remains largely unexplored.

To investigate the prenatal events driving disease development, we here developed and utilized a novel inducible murine *KMT2A::AFF1* model, enabling precise targeting of the fusion gene to hematopoietic stem and progenitor cells. This approach uncovered a distinct fetal pre-leukemic state and identified a transcriptional signature transferable to human *KMT2A::AFF1* leukemia. Our findings provide critical insights into the ontogeny and cell-type-specific effects of KMT2A::AFF1, and the transcriptional alterations underlying the pre-leukemic state.

## Methods

Please see *supplementary methods* for details.

### Animals

*Col1a1-tetO-KMT2A::AFF1* (*KMT2A::AFF1*^*Col1a1-tetO*^), *Rosa26-ZtTA* [19] and different Cre strains were used to enable cell type specific induction of *KMT2A::AFF1* (Vav-Cre [20]; Rag1-Cre [21]; Mb1-Cre [22]). Embryos were obtained by timed mating overnight. Adult mice were analyzed 8–30 weeks of age if not otherwise stated. All animal experiments were performed in accordance with the relevant guidelines and regulations and were approved by the Malmö/Lund Ethics Committee on Animal Testing at the Lund District Court.

### Colony-forming assays

In vitro semi solid cultures were performed with ~100-250 purified cells that were cultured in Methocult (Stemcell Technologies) supplemented with cytokines. Colonies were scored and replated weekly.

### Transplantation assay

Recipient NOD-SCID-GAMMA (Il2rg^–/–^) (NSG) mice (CD45.1) were sub-lethally irradiated (250 cGy) and donor CD24^+^ PreProBs cells ( ~ 500–1300cells/recipient) or LSK (Lin^-^; SCA1^+^; cKIT^+^) cells ( ~ 1500–2000 cells/ recipient) were purified using Fluorescence-Activated Cell Sorting (FACS) and subsequently transplanted.

### Single-cell cellular indexing of transcriptomes and epitopes by sequencing

Cells were purified and loaded onto the Chromium platform Next GEM Single Cell 3’ Kit v3.1 (10x Genomics) and subjected to library preparation, according to the manufacturer’s instruction [23]. The resulting libraries were sequenced on a NovaSeq system (Illumina).

### Data analysis

Flow cytometry data were analyzed using FlowJo_v10 (BD). Graphs and statistical analysis were performed using GraphPadPrism_v10. Single-cell RNA-sequencing data were visualized in ShinyCell [24].

## Results

### KMT2A::AFF1 induction in embryonic hematopoietic stem and progenitor cells

**Fig. 1:**
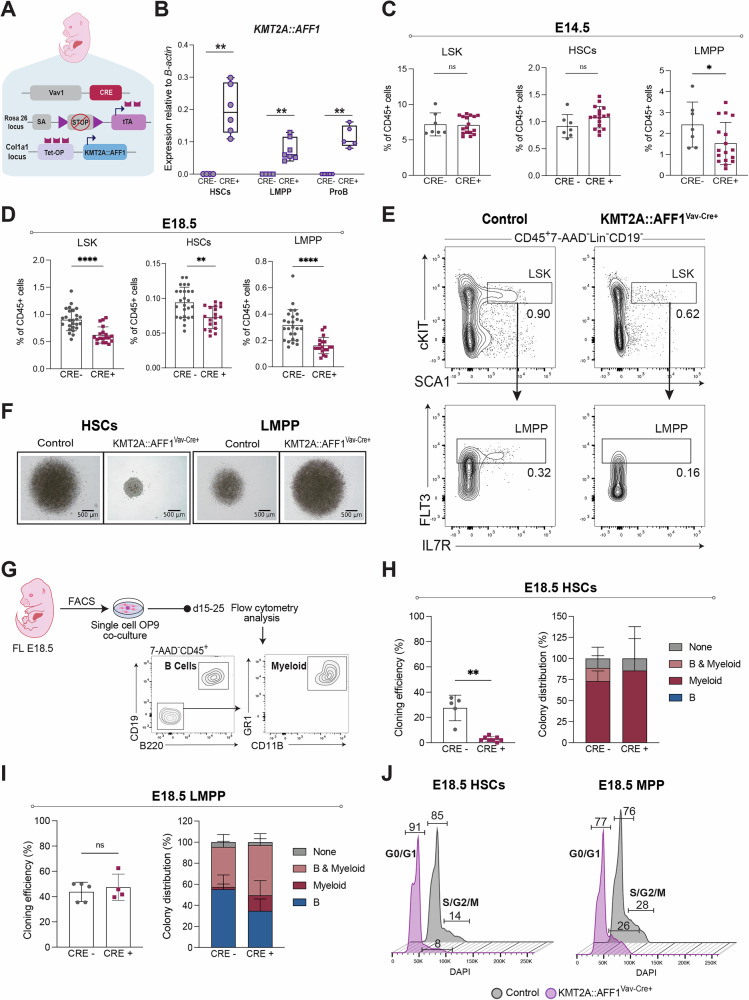
*KMT2A::AFF1 impacts proliferation and lineage output of fetal HSPCs.* **A** Schematic illustration of the model. Human *KMT2A::AFF1* was placed under the control of a Tet-OP promotor; the tissue recombinase (tTA) had a STOP cassette flanked by LoxP sites. Strain specific Cre-recombinase was used to induce expression of *KMT2A::AFF1*. **B** Relative expression (normalized to *B-actin)* of *KMT2A::AFF1* in purified HSCs, LMPPs and ProBs from E18.5 *KMT2A::AFF1*^Vav-Cre+^ fetal livers (FLs). Box plots define lower and upper quartiles, and whiskers min to max values (4 FACS experiments). **C**, **D** Frequencies of LSK, HSCs and LMPPs as percent of CD45^+^ cells in control and *KMT2A::AFF1*^Vav-Cre+^ FLs at (**C**) E14.5 (3 experiments) and (**D**) E18.5 (6 experiments). **E** Flow cytometry plots of LSK compartment in control and *KMT2A::AFF1*^Vav-Cre+^ FLs at E18.5. Cells were gated CD45^+^Lin^–^CD19^–^. Further gating is indicated in the figure showing LSK (*top*) and LMPPs (*bottom*). Numbers are mean percentages of total CD45^+^ cells. **F** Representative photos of E18.5 HSCs (*left*) and LMPPs (*right*) bulk liquid cultures at day 7. Genotypes (control and *KMT2A::AFF1*^Vav-Cre+^) are indicated. **G** Schematic illustration of single-cell OP9 co-culture workflow and subsequent evaluation of B and myeloid linage potentials using flow cytometry. **H**, **I** Single HSCs (**H**) and LMPPs (**I**) were co-cultured on OP9 stroma. Frequency of colonies (*left*) and linage output (*right*) for control and *KMT2A::AFF1*^Vav-Cre+^ FLs at E18.5 are shown. Colonies were defined as B, myeloid; B and myeloid (B & My) or not designated to any of these (none) (3 experiments). **J** Cell cycle analysis of HSCs (*left*) and MPPs (LSKCD150^-^CD48^+^)(*right*) at E18.5. Mean percentages of cells in G0/G1 and S/G2/M for each population are shown (1–2 embryos per genotype,1 experiment). Bars show means ± SD, and each dot represents an individual embryo. *p ≤ 0.05; ** *p* ≤ 0.01; ****p ≤ 0.0001; n.s. not significant.

To study the KMT2A::AFF1-associated pre-leukemic state, we created a novel inducible mouse model. For this, we placed a human *KMT2A::AFF1* fusion gene under the control of a Tet-op promoter (*KMT2A::AFF1*^Col1a1-tetO^), as described [25]. The fusion was made Cre inducible by crossing with the *Rosa26-ZtTA* strain [19], in which the tetracycline-trans-activator (tTA) contains a STOP cassette flanked by LoxP sites. Subsequently, we induced the expression of *KMT2A::AFF1* in embryonic hematopoietic stem and progenitor cells (HSPCs) using Vav-Cre recombinase [20], resulting in *KMT2A::AFF1*^*Col1a1-tetO /ZtTA*/Vav-Cre+^ strain (named *KMT2A::AFF1*^Vav-Cre+^ throughout) (Fig. 1A). Cre-positive FLs from embryonic day (E)18.5 expressed *KMT2A::AFF1* at ~2.5 to 4.5-fold higher levels than endogenous *Kmt2a*, and Homeobox A9 (*Hoxa9*), a known *KMT2A::AFF1* target gene [26], was significantly elevated (Fig. 1B**;** Supplementary Fig. 1A, B).

We observed that *KMT2A::AFF1*^Vav-Cre+^ pups were born at slightly lower Mendelian ratios than littermate controls. They were also smaller in size and did not survive beyond the neonatal period. The spleens of these pups were significantly reduced and contained less mature lymphoid and erythroid cells, whereas the number of myeloid CD11B^+^GR1^+^ cells were comparable to controls (Supplementary Fig. 1C–E). In addition, HSPCs were clearly affected, with the LSK compartment reduced almost twofold (LSK; negative for mature lineage markers Lin^-^; SCA1^+^; cKIT^+^) (Supplementary Fig. 1F).

Thus, *KMT2A::AFF1* induction with Vav-Cre recombinase triggered a postnatal hematopoietic phenotype affecting HSPCs as well as more mature blood cell lineages.

### KMT2A::AFF1 impacts proliferation and lineage output of HSPCs

Human KMT2A::AFF1 disease initiation is recognized to occur *in utero*; therefore, we next explored the consequences of *KMT2A::AFF1* during embryogenesis. As a window of susceptibility to *KMT2A::AFF1* has been suggested between E12-14 in mice [16], FLs from E14.5 were investigated. Here we observed a mild phenotype within the HSPC compartment, mainly seen as a reduction in lympho-myeloid primed progenitors (LMPPs; LSKFLT3^+^) [27] (Fig. 1C**;** Supplementary Fig. 2A). As the phenotype was mild compared to what was seen postnatally, later fetal stages were investigated. At E18.5, a more pronounced effect of the oncogene was seen, with a reduction in the LSK compartment, both in frequency and absolute number, along with a continued decrease in LMPPs, as seen at E14.5 (Fig. 1D, E; Supplementary Fig. 2B, C). Functionally, these LMPPs proliferated similar to control cells in vitro, whereas *KMT2A::AFF1*^*+*^ HSCs (LSKFLT3^-^CD150^+^CD48^-^) [28, 29] were found to expand less than controls (Fig. 1F). Additional experiments evaluating proliferation and lineage output at the single-cell level were therefore performed. In agreement with the results from the bulk cultures, a significant reduction in colony formation of single *KMT2A::AFF1*^*+*^ HSCs compared to control was observed (2.9% vs 27.5%) (Fig. 1G, H). In the colonies produced from *KMT2A::AFF1*^*+*^ HSCs, B lineage output was sparse, and clones were biased towards the myeloid lineage (Fig. 1H). No difference in colony formation was observed between *KMT2A::AFF1*^*+*^ LMPPs and control cells, however, as observed for HSCs, lineage output was skewed towards the myeloid lineage (Fig. 1I). In agreement with our in vitro data, cell cycle analysis showed that *KMT2A::AFF1*^+^ HSCs had a higher proportion of cells in G0/G1 compared to control (91% vs 85%) (Fig. 1J).

Together, our findings show that *KMT2A::AFF1* exerts differential effects across early hematopoietic populations. While HSC proliferation was impaired, LMPPs retained their colony-forming capacity. Both populations, however, exhibited a bias towards myeloid output compared to controls.

### A unique CD24^+^ PreProB population emerges upon KMT2A::AFF1 induction in embryonic HSPCs

**Fig. 2:**
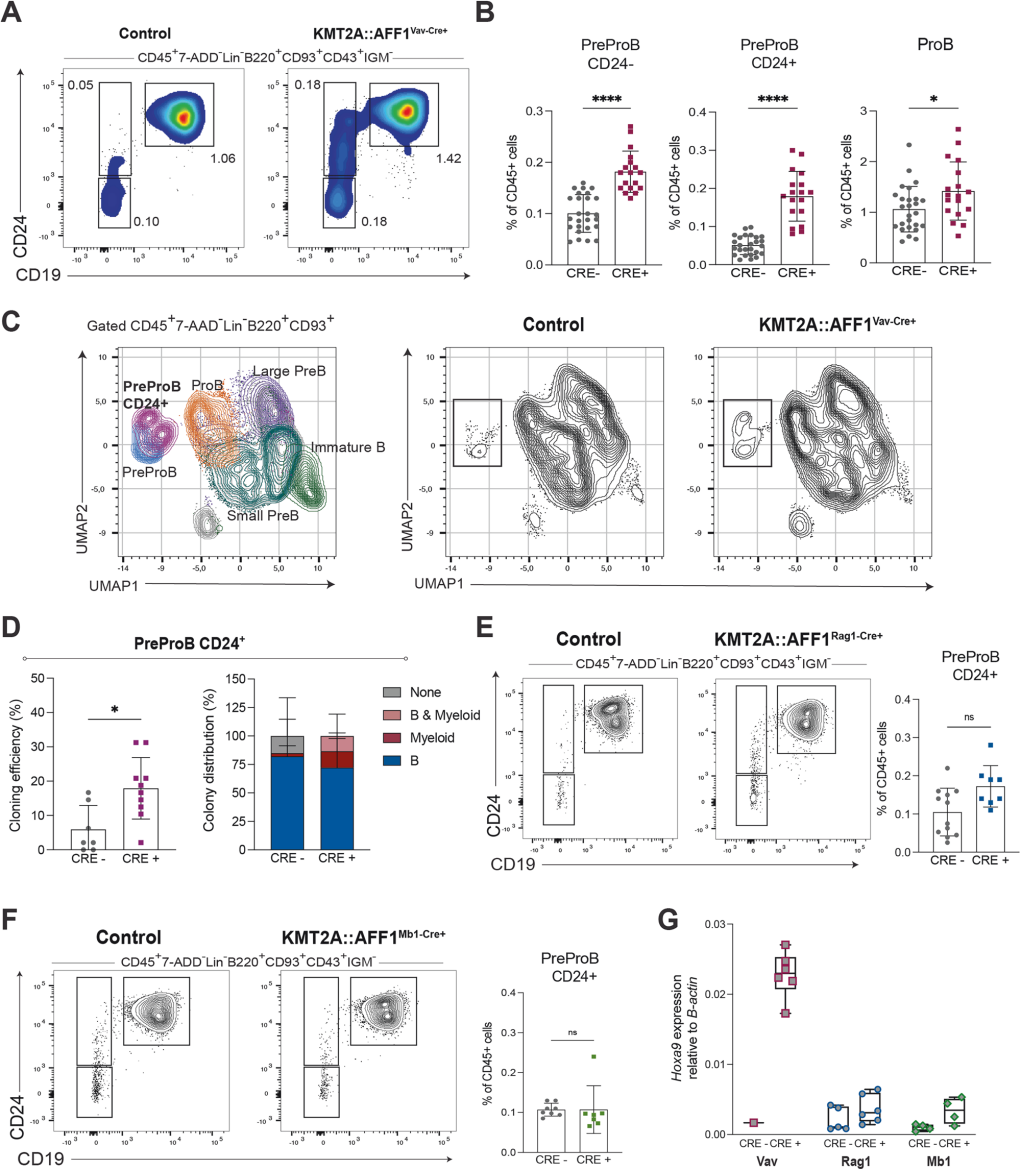
A unique CD24^+^ PreProB population emerges upon *KMT2A::AFF1* induction in embryonic HSPCs. **A** Flow cytometry plots of B progenitor compartment in control and *KMT2A::AFF1*^Vav-Cre+^ embryos at E18.5. Numbers show mean percentages of total CD45^+^ cells. **B** Frequencies of B progenitors as percentage of CD45^+^ cells in control and *KMT2A::AFF1*^Vav-Cre+^ FLs at E18.5 (6 experiments). **C** UMAP visualizations of flow cytometry data from control and *KMT2A::AFF1*^Vav-Cre+^ embryos, displaying different B progenitors in E18.5 FLs. Combined UMAP is shown to the left, and per genotype to the right (3–5 embryos/genotype, down sampled to 10.000cells/embryo, one experiment). **D** Single CD24^+^ PreProBs were co-cultured on OP9 stroma. Frequency of colonies (*left*) and linage output (*right*) for control and *KMT2A::AFF1*^Vav-Cre+^ FLs at E18.5 are shown. Colonies were defined as B (CD19^+^B220^+^), myeloid (CD11B^+^GR1^+^); B and myeloid (B & My) or not described to any of these (none) (3 experiments). **E**, **F** B progenitor compartment in control and (**E**) *KMT2A::AFF1*^Rag1-Cre+^ or (**F**) *KMT2A::AFF1*^Mb1-Cre+^ embryos at E18.5. Flow cytometry plots (*left*) and frequencies of CD24^+^ PreProBs as percentage of CD45^+^ cells (*right*) (3–4 experiments). **G** Relative expression (normalized to *B-actin)* of *Hoxa9* in purified E18.5 CD24^+^ PreProBs from controls and *KMT2A::AFF1* Vav-Cre; Rag1-Cre and Mb1-Cre respectively. Box plots define lower and upper quartiles, and whiskers min to max values. (*KMT2A::AFF1*^Rag1-Cre+^ embryos were heterozygous or homozygous for *KMT2A::AFF1*^*Cola1-tetO*^.) Bars show means ± SD and each dot represents an individual embryo. *p ≤ 0.05; ****p ≤ 0.0001. n.s. not significant.

We next explored the impact of the *KMT2A::AFF1* oncogene on the B progenitor compartment at E18.5 [30, 31] (gating strategy in Supplementary Fig. 3A). Using flow cytometry, both PreProB (B220^+^CD43^+^CD19^-^) and ProB (B220^+^CD43^+^CD19^+^IGM^-^cKIT^+^) progenitors were significantly increased in *KMT2A::AFF1*^+^ embryos compared to littermate controls. Furthermore, within the B220^+^CD43^+^ population, cells with a CD24^+^CD19^-^ phenotype were observed; a population hardly detected in the control animals (Fig. 2A, B; Supplementary Fig. 3B). CD24 is a surface marker that is typically dim on PreProB progenitors and is first upregulated at the CD19^+^ ProB cell stage [30, 31]. When the B220^+^CD93^+^ compartment was visualized on a flow cytometry UMAP, the *KMT2A::AFF1* PreProBs clustered with control, despite disparities in CD24 expression (Fig. 2C). The majority of the *KMT2A::AFF1*^*+*^ PreProB cells (independent of CD24 status) also had surface expression of the progenitor marker cKIT, which was lower on control cells (Supplementary Fig. 3C). Of note, an increase in CD24^+^ PreProB cells could be observed already at E14.5 (Supplementary Fig. 3D). Functionally, the *KMT2A::AFF1* CD24^+^ PreProBs at E18.5 displayed an increased cloning capacity (6% vs 18%, respectively) and notable lineage plasticity, giving rise to both B and myeloid colonies, whereas control cells predominantly generated B cell colonies (Fig. 2D).

In an earlier study, using a different murine *KMT2A::AFF1* model, long latency lymphomas developed when the fusion was induced in lymphoid progenitors [14]. Thus, we sought to assess whether the CD24^+^ PreProB population would emerge in our model if *KMT2A::AFF1* was induced in lymphoid committed progenitors in the embryo. Rag-1 (Recombination activating gene 1) is involved in VDJ recombination and expressed in lymphoid progenitors in adults, and lympho-myeloid committed progenitors in the embryo [32, 33], whereas Mb1 (also named Cd79a) is part of the B cell antigen receptor. Hence, *KMT2A::AFF1* expression was induced using the Rag1-Cre [21] and Mb1-Cre [22] strains (Supplementary Fig. 4A, B). Analysis of the PreProB compartment at E18.5 showed no significant difference between *KMT2A::AFF1*^*+*^ and control cells at this stage and *Hoxa9* expression was only marginally increased compared to control (Fig. 2E–G; Supplementary Fig. 4C–F). Thus, a unique CD24^+^ PreProB population emerges specifically upon *KMT2A::AFF1* induction in HSPCs.

### B progenitor compartment is unaffected by postnatal induction of KMT2A::AFF1

**Fig. 3:**
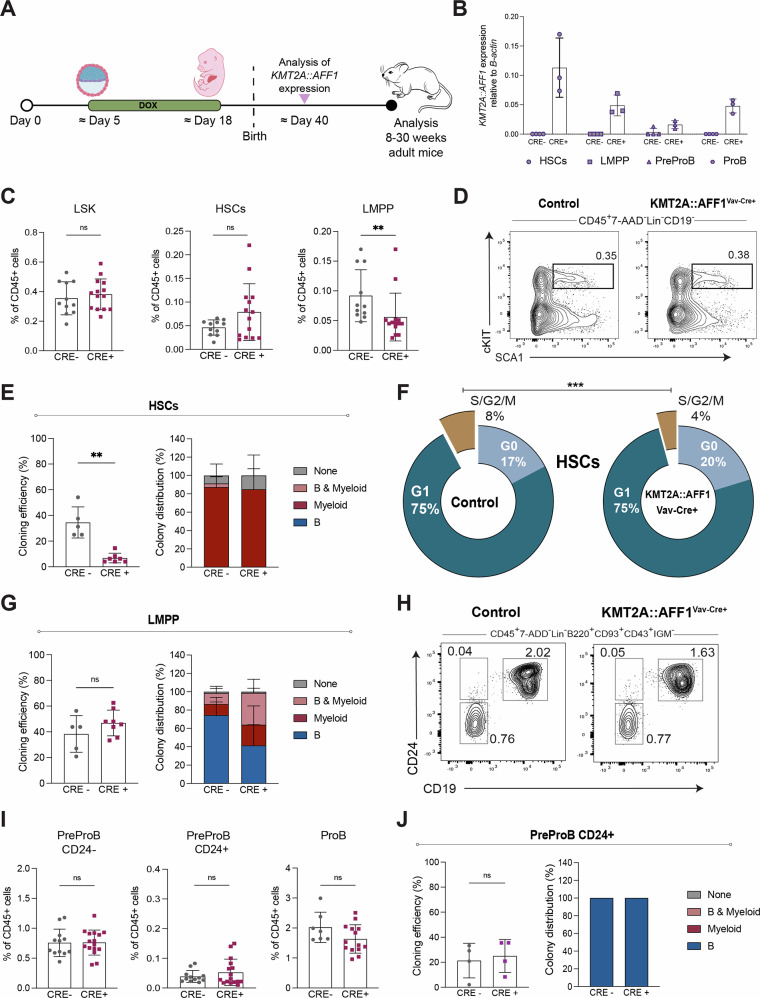
B progenitor compartment is unaffected by postnatal induction of *KMT2A::AFF1.* **A** Schematic illustration of postnatal induction of *KMT2A::AFF1* by administration of Doxycycline (Dox) food to the female from approx. day 5 to 18 of pregnancy. *KMT2A::AFF1* expression was analyzed after ∼6-weeks and phenotype at 8–30 weeks. **B** Relative expression (normalized to *B-actin)* of *KMT2A::AFF1* in purified HSCs, LMPPs, PreProBs (CD24^-/+^) and ProBs in bone marrows (BMs) from ∼6-week-old mice, treated with Doxycycline as shown in panel (**A**) (1 FACS experiment). **C** Frequencies of LSK, HSCs and LMPPs as percent of CD45^+^ cells in control and *KMT2A::AFF1*^Vav-Cre+^ postnatally induced mice (6 experiments, *n*_males_ = 7; *n*_females_ = 18). **D** Flow cytometry plots of LSK compartment in control and *KMT2A::AFF1*^Vav-Cre+^ postnatally induced mice. Numbers are mean percentages of total CD45^+^ cells. **E** Single HSCs were co-cultured on OP9 stroma. Frequency of colonies (*left*) and linage output (*right*) for control and *KMT2A::AFF1*^Vav-Cre+^ postnatally induced mice are shown. Colony distribution defined as B (CD19^+^B220^+^), myeloid (CD11B^+^GR1^+^); B and myeloid (B & My) or not described to any of these (none) (3 experiments). **F** Cell cycle analysis of HSCs from control and *KMT2A::AFF1*^Vav-Cre+^ postnatally induced mice. Mean percentages of cells in G0, G1 and S/G2/M are shown (6–8 mice per genotype, 3 experiments). **G** Single LMPPs were co-cultured on OP9 stroma. Frequency of colonies (*left*) and linage output (*right*) for control and *KMT2A::AFF1*^Vav-Cre+^ postnatally induced mice are shown. Colony distribution defined as in panel (**E**) (5 experiments). **H** Flow cytometry plots of B progenitor compartment in control and *KMT2A::AFF1*^Vav-Cre+^ postnatally induced mice. Numbers show mean percentages of total CD45^+^ cells. **I** Frequencies of B progenitors as percentage of CD45^+^ cells in control and *KMT2A::AFF1*^Vav-Cre+^ postnatally induced mice (7 experiments, *n*_males_ = 8; *n*_females_ = 13–20) (ProB not defined by cKIT expression). **J** Single CD24^+^ PreProBs were co-cultured on OP9 stroma. Frequency of colonies (*left*) and linage output (*right*) for control and *KMT2A::AFF1*^Vav-Cre+^ postnatally induced mice are shown. Colony distribution as defined in panel E (3 experiments). Bars show means ± SD and each dot represents an individual mouse. **p ≤ 0.01; ***p ≤ 0.001; n.s. not significant.

To explore the consequences of a postnatal induction of the oncogene, pregnant females were given doxycycline (dox) to delay the induction of *KMT2A::AFF1* (Fig. 3A). Expression of *KMT2A::AFF1* was analyzed after birth and confirmed at ∼ day 40. In adult mice ( > 8 weeks) a mild phenotype was observed, including a reduction in LMPPs, whereas HSCs were unaffected (Fig. 3B–D; Supplementary Fig. 5A). Functionally, *KMT2A::AFF1* induced myeloid bias in both HSCs and LMPPs, and *KMT2A::AFF1*^*+*^ HSCs formed fewer colonies, and a smaller proportion of cells were actively cycling (Fig. 3E–G). Thus, the phenotype observed in the LSK compartment in postnatally induced mice resembled that of their embryonically induced counterparts.

However, when we assessed the B progenitor compartment in these mice, it was largely unaffected, and specifically no expansion of the CD24^+^ PreProB subset was observed (Fig. 3H–I; Supplementary Fig. 5B, C). The few KMT2A::AFF1 CD24^+^ PreProB cells seen were purified and analyzed in single-cell stromal co-cultures. However, their cloning capacity and lineage output were comparable to those of their control counterparts (Fig. 3J).

These findings highlight a developmental susceptibility to the oncogene, with the observed expansion of CD24^+^ PreProBs strictly dependent on *KMT2A::AFF1* expression in embryonic HSPCs.

### Embryonic CD24^+^ PreProBs emerging in response to KMT2A::AFF1 display self-renewal capacity

**Fig. 4:**
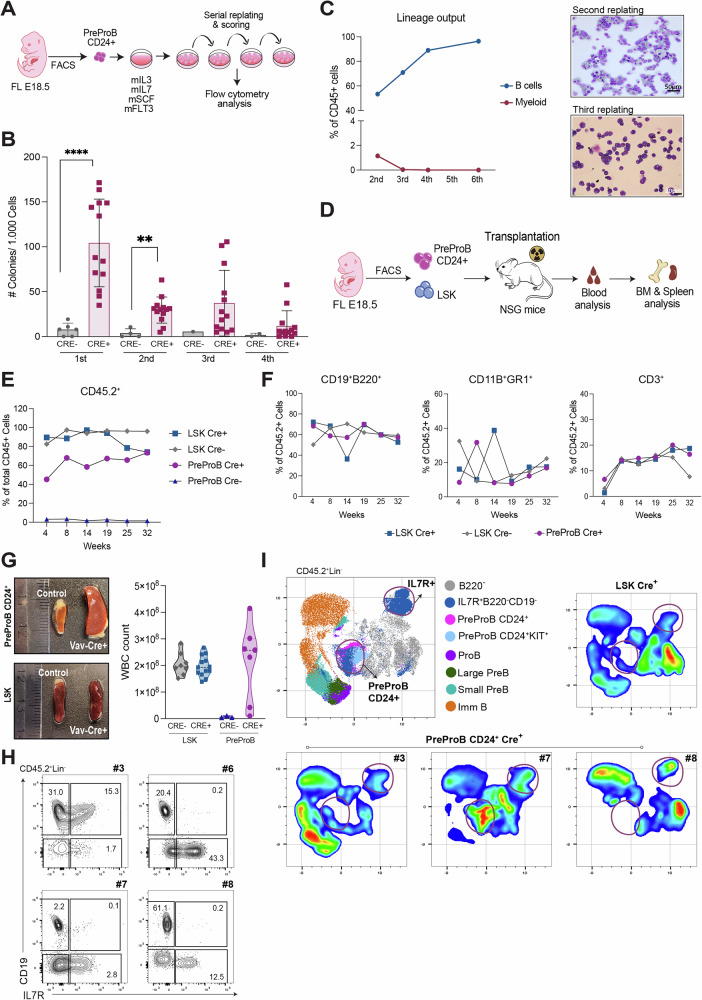
Embryonic CD24^+^ PreProBs emerging in response to *KMT2A::AFF1* display self-renewal capacity. **A** Schematic illustration of the replating assay in semi-solid media. **B** Semi-solid in vitro cultures of CD24^+^ PreProBs control and *KMT2A::AFF1*^Vav-Cre+^ fetal livers (FLs) from E18.5. Scoring and replating was done weekly. Total number of colonies/1.000 cells for individual embryos are shown. Means ± SD (4 experiments). **C** Frequencies of **B** (CD19^+^B220^+^) (*blue*) and myeloid (CD11B^+^GR1^+^) (*red*) cells detected by flow cytometry at different time points in the replating culture of *KMT2A::AFF1*^Vav-Cre+^ CD24^+^ PreProBs. Mean percentages of total CD45^+^ cells (*left*) and representative cytospin *(right)* of cells at 2^nd^ *(top)* and 3^rd^ *(bottom)* replating. **D** Schematic illustration of in vivo assay. **E**, **F** LSK and CD24^+^ PreProBs from control and *KMT2A::AFF1*^Vav-Cre+^ were transplanted into sub-lethally irradiated NSG mice and donor cells assessed in peripheral blood at the indicated time-points. **E** Mean frequencies of CD45.2 donor cells displayed as percentages of total CD45^+^ cells. **F** Lineage distribution (B/ Myeloid/ T) of reconstituted *KMT2A::AFF1*^*+*^ PreProBs and LSK mice shown as mean percentages of total CD45.2^+^ cells (n_LSK_=  1–3; n_PreProB_ = 1–9). **G** Photos (*left*) and violin plot of white blood cell (WBC) counts (*right)* of recipient´s spleens at final readout, 2-8 months after transplantation. Immunophenotype (CD24^+^ PreProBs or LSK) and genotype of donor cells are indicated. **H** Immunophenotype of recipient´s bone marrow (BM) transplanted with CD24^+^ PreProBs from *KMT2A::AFF1*^Vav-Cre+^ embryos. Cells were gated for donor CD45.2^+^ cells and negative for lineage markers. CD19 and IL7R are displayed for individual mice. Numbers show percentage of total CD45.2^+^ cells. **I** UMAP visualizations of flow cytometry data from transplanted *KMT2A::AFF1*^Vav-Cre+^ embryos, displaying different B progenitors. Combined UMAP of CD45.2^+^7-AAD^-^Lin^-^ cells (4 PreProBs, 2 LSKs) (*top, left*), one *KMT2A::AFF1*^+^ LSK (*top, right*) and three different *KMT2A::AFF1*^+^ CD24^+^ PreProB recipients (*bottom*). Down sampled to 30.000 cells/mouse. **p ≤ 0.01; ****p ≤ 0.0001.

The unique PreProB population observed in the embryo could potentially represent leukemia initiating cells. As such, the cells should possess self-renewal capacity, which was subsequently investigated by serial replating in a semi-solid culture assay (Fig. 4A). After one week of culture the *KMT2A::AFF1*^+^ expressing CD24^+^ PreProBs produced significantly more colonies than control cells and *KMT2A::AFF1*^+^ cells were successfully replated for more than four rounds (Fig. 4B). The colonies were primarily of B (B220^+^CD19^+^) phenotype, but some myeloid (CD11B^+^GR1^+^) cells were also detected during the first weeks of culture (Fig. 4C). Cells with an immunophenotype compatible with CD24^+^ PreProBs (B220^+^CD43^+^CD24^+^CD19^-^) were observed during the early replating rounds but gradually decreased over time, consistent with a decline in colony numbers (Supplementary Fig. 6A).

The observed self-renewal capacity in vitro led to further investigations in vivo. Since the *KMT2A::AFF1*^Vav-Cre+^ mice did not survive the postnatal period, these investigations were conducted in an adult transplantation setting. NOD-SCID-GAMMA (Il2rg^-/-^) (NSG) mice were used as recipients, as these lack lymphoid cells, allowing for donor B cells to expand without competition. LSKs and CD24^+^ PreProBs from *KMT2A::AFF1*^*+*^ and control embryos were subsequently transplanted into sub-lethally irradiated NSG mice (Fig. 4D). None of the control PreProB recipients showed any long-term reconstitution in peripheral blood, whereas a majority of the *KMT2A::AFF1*^*+*^ CD24^+^ PreProB transplanted mice displayed long-term engraftment, with levels close to those obtained with LSKs (Fig. 4E). Reconstituted *KMT2A::AFF1*^*+*^ CD24^+^ PreProB mice also displayed multilineage engraftment, with donor-derived B (CD19^+^), T (CD3^+^) and myeloid (CD11B^+^GR1^+^) cells detected in peripheral blood (Fig. 4F). High engraftment levels were also observed in the BM (5 out of 7 analyzed mice; Supplementary Fig. 6B) and several of these mice had enlarged spleens (Fig. 4G).

Additionally, despite transplanting an immunophenotypically strict population, the CD24^+^ PreProB reconstituted mice exhibited diverse phenotypes. Some displayed an increase in an interleukin 7 receptor (IL7R) expressing population, that was negative for mature lymphoid markers (CD19, CD3, NK1.1) as highlighted in an unsupervised UMAP (Fig. 4H–I; Supplementary Fig. 6C). IL7R is known to define common lymphoid progenitors in mice, and essential for the development of B and T cells [34–36]. Hence, these mice exhibit a phenotype indicative of a more immature lymphoid population. Of note, mice #6 and #8 displayed signs of illness and were sacrificed prematurely at 2 and 5 months respectively. Mouse (#6) had low engraftment levels in the bone marrow (4.6%). Despite this, the Lin^-^CD19^-^IL7R^+^ population was observed. Some mice also displayed a partial block in B cell differentiation, and mouse #7 had an aberrant CD24^+^ PreProB population (Supplementary Fig. 6D). While the immature lymphoid population observed is reminiscent of findings in a previous murine ALL model [37], we did not observe any differentiation block in the mice transplanted with *KMT2A::AFF1*^+^ LSK cells.

Taken together, our results demonstrate distinct properties of the oncogene in different cell populations. Notably, the PreProB subset, marked by surface CD24 expression, exhibits self-renewal capacity and reduced differentiation potential, suggesting a role as leukemia-initiating cells.

### KMT2A::AFF1 induces transcriptional changes in fetal and adult HSPCs

**Fig. 5:**
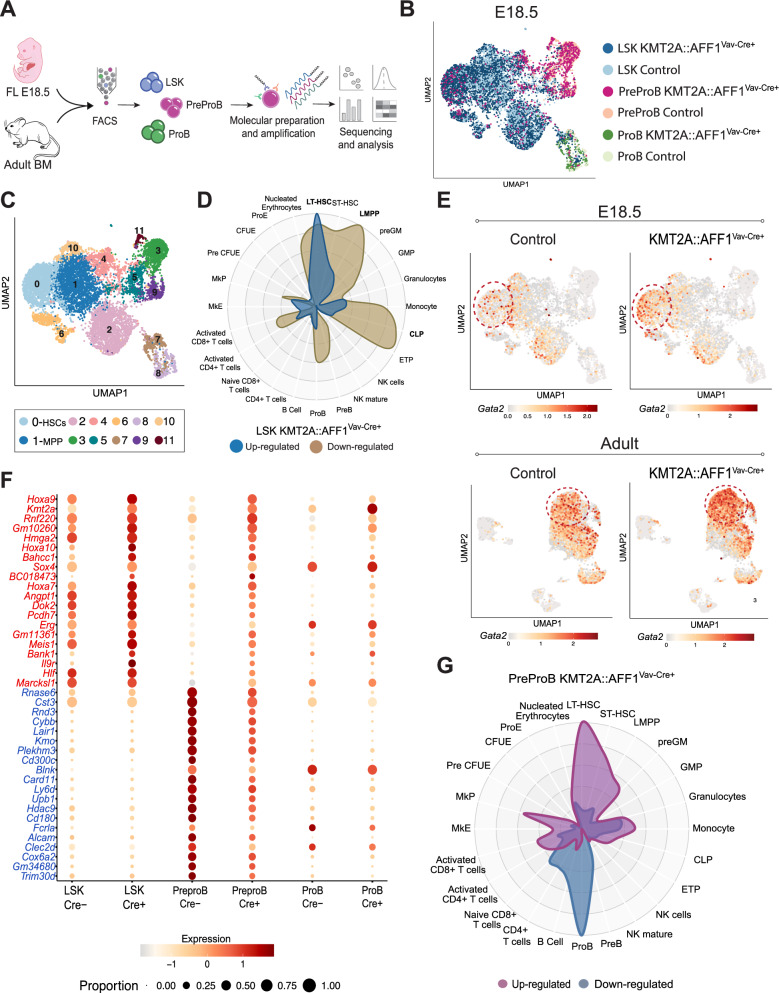
*KMT2A::AFF1* induces transcriptional changes in fetal and adult HSPCs and fetal PreProBs. **A** Schematic illustration of the single-cell RNA-seq experimental workflow. **B**, **C** UMAPs of LSK, PreProB and ProB cells from control and *KMT2A::AFF1*^Vav-Cre+^ E18.5 embryos. **B** Color coded based on purified population and genotype and (**C**) based on cluster. **D** Lineage affiliation of shared up- (*blue*) and down-regulated (*brown*) DEGs in *KMT2A::AFF1*^Vav-Cre+^ LSKs compared to control, analyzed with CellRadar (*p* adj<0.01; |log2 foldchange | >0.5). **E** UMAPs of LSK, PreProBs and ProB cells from control and *KMT2A::AFF1*^Vav-Cre+^ E18.5 embryos (*top*) and adult (*bottom*), highlighting *Gata2* expressing LSK cells. Down sampled to equal cell number. **F** Dot plot displaying the top up- and down-regulated DEGs in *KMT2A::AFF1*^Vav-Cre+^ PreProBs compared to control at E18.5 for all purified populations. Red indicates up- and blue down-regulated genes. **G** Lineage affiliation of significantly up- (*purple*) and down-regulated (*blue*) DEGs in *KMT2A::AFF1*^Vav-Cre+^ PreProBs compared to control at E18.5, analyzed with CellRadar (*p* adj < 0.01; |log2 foldchange | >1.0).

To assess the molecular impact of *KMT2A::AFF1* induction, multimodal single-cell RNA-sequencing (RNA-seq) using CITE-seq (cellular-indexing-of-transcriptomes-and-epitopes) was performed [23]. The analysis included PreProB cells (irrespective of CD24 expression), as well as LSKs and ProB cells from both E18.5 embryos and postnatally induced adult mice (Fig. 5A–C; Supplementary Fig. 7A–C; Supplementary Table 1). The UMAPs, divided by developmental stage, had distinct cell states based on expression of established marker genes such as *Mllt3, Il7r* and *Mpo* (Fig. 5C; Supplementary Fig. 7C–F).

The most striking functional consequences of the oncogene in the HSPC compartment were a general myeloid bias and a reduced cloning capacity of HSCs, and these phenotypes were independent of developmental states of induction (Fig. 1H–J; Fig. 3E–G). We therefore extracted developmentally shared differentially expressed genes (DEGs) in *KMT2A::AFF1*^*+*^ LSKs compared to control (Supplementary Fig. 7G; Supplementary Table 2). The up-regulated shared DEGs were associated with ‘LT-HSC’, whereas the down-regulated genes were linked to a more lymphoid signature, in agreement with the myeloid bias observed functionally (Fig. 5D). Among the joint up-regulated genes we also observed the transcription factor *Gata2* (Fig. 5E; Supplementary Table 2), which might be relevant as increased activity of *Gata2* induces quiescence of HSCs in both mice and human [38]. Thus, the molecular landscape of the HSPC compartment underscores the impact of *KMT2A::AFF1* on HSCs proliferation, as well as its general effect on lineage affiliation, regardless of developmental state.

### Embryonic KMT2A::AFF1^+^ PreProBs display a pre-leukemic stem cell signature

Subsequently, we compared *KMT2A::AFF1*^*+*^ PreProBs and ProBs from fetal and adult stages to their age-matched normal counterparts. The fetal PreProB stood out as most different to normal counterparts with >250 DEGs (*p* adj<0.01; |log2 fold change | >1.0; Supplementary Fig. 7H). *KMT2A::AFF1*^*+*^ target genes, such as *Hoxa9* and *Meis1* were highly expressed [26], and genes associated with stemness such as *Hlf* and *Mecom* (*Evi1*) were up-regulated [39, 40] (Fig. 5F; Supplementary Fig. 7H**)**. Furthermore, the up-regulated genes were predominantly affiliated with ‘LT-HSC’ while the down-regulated genes were more closely associated with ‘ProB’ (Fig. 5G), together indicative of a differentiation block.

**Fig. 6:**
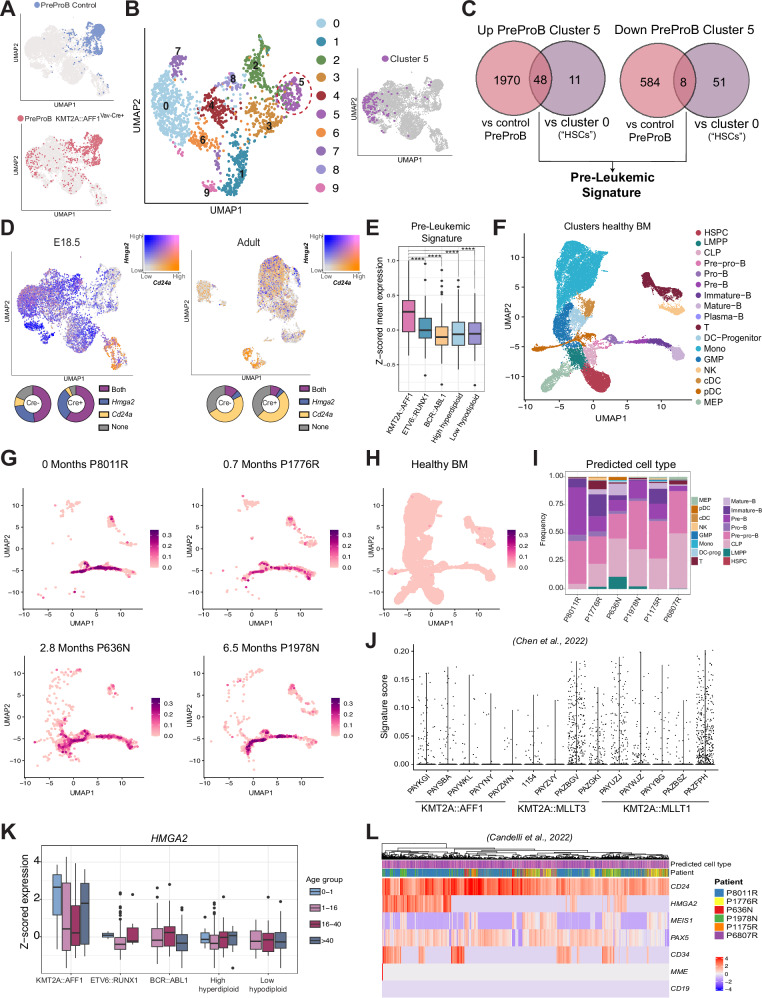
The pre-leukemic *KMT2A::AFF1* signature is transferable to leukemic patient samples. **A** UMAPs of LSK, PreProBs and ProBs from control and *KMT2A::AFF1*^Vav-Cre+^ embryos, highlighting PreProBs in control (*top*) and *KMT2A::AFF1*^Vav-cre+^ (*bottom*), respectively. **B** UMAP of PreProBs from control and *KMT2A::AFF1*^Vav-Cre+^ embryos, with the different clusters (*left*) and displaying the cells from cluster #5 on the UMAP from Fig. 5B (*right*). **C** Venn diagram of up- and downregulated genes in cluster #5 compared to control PreProBs and control cells in cluster #0 from the combined UMAP (“molecular HSC cluster”). The universal up- and downregulated genes formed the pre-leukemic signature. **D** UMAP displaying co-expression of *Hmga2* and *Cd24a* in *KMT2A::AFF1* expressing cells at E18.5 (*left*) and in postnatally induced mice (*right*). Pie charts show percentage of cells expressing each gene in Cre^+^ and Cre^-^ cells, respectively. **E** Boxplot showing z-scored mean expression of the up-regulated genes from the pre-leukemic signature in different subtypes of ALL (*n* = 67–254) from bulk RNA-seq samples [46]. The box includes first to third quartile and the line indicates the median. **F** UMAP of healthy BM [48]. **G** Expression of the pre-leukemic signature in individual KMT2A::AFF1 infant ALL samples [47], mapped onto the UMAP in panel (**F**). Color scale set to the same range for all samples (0.35). Ages of the patients are listed on top. **H** Lack of expression of the pre-leukemic signature in the healthy BM reference. **I** Bar plots displaying predicted cell type (from healthy BM in **F**) of the signature positive cells in KMT2A::AFF1 leukemic patient samples [47]. **J** Violin plot displaying the UCell score of the pre-leukemic signature among different KMT2A rearranged infant ALLs [48]. **K** Boxplot of *HMGA2* expression in different subtypes of ALL (*n* = 67–254) [46] displayed per age group. The box includes first to third quartile and the line indicates the median. **L** Heatmap displaying expression of selected genes in *CD24*^*+*^*CD19*^*-*^ cells from KMT2A::AFF1 infants [47]. ****p adj  ≤ 0.0001.

We next visualized the PreProBs alongside LSK and ProB cells in the combined UMAP. Adult PreProBs, regardless of *KMT2A::AFF1*^*+*^ status, and fetal control cells all clustered together. In contrast, a fraction of the fetal *KMT2A::AFF1*^*+*^ PreProBs clustered with molecularly more primitive cells within the LSK compartment (Fig. 6A**;** Supplementary Fig. 8A).

Due to the molecular heterogeneity seen, a separate UMAP displaying only PreProBs was generated. Specifically, one cluster in this UMAP (#5) contained primarily *KMT2A::AFF1*^*+*^ cells. When assessed in the combined UMAP, these cells mapped to the primitive cells and importantly expressed *Cd24a* (Fig. 6B; Supplementary Fig. 8B, C), linking it to the expanded unique population seen by flow cytometry.

To obtain the molecular signature of the pre-malignant cells, the PreProB cluster #5 cells were compared to their normal PreProB counterparts as well as to the control cells in cluster 0 (molecular ‘HSCs’) from the combined UMAP (Fig. 5C). The comparison rendered 48 commonly up-regulated genes, including KMT2A::AFF1 target genes such as *Meis1* and *Hoxa* genes (Fig. 6C; Supplementary Table 3). As the murine pre-leukemic clone was unique to ontogeny, we assessed the signature for genes characteristic to fetal hematopoiesis. One such gene is high-mobility-group-AT-hook-2 *(Hmga2)*, which is higher expressed during ontogeny in both mouse and human [41–44]. It has also been reported as a direct target of KMT2A::AFF1 [26, 45]. As the expanded PreProB population was defined by surface expression of CD24, the relationship between *Hmga2* and *Cd24a* was assessed. We found that the genes were largely co-expressed in the embryo (48-59%), in contrast to adults, where only a small fraction of the cells (9-12%) co-expressed the genes (Fig. 6D).

Collectively, these data define the transcriptional signature of the pre-leukemic cells and links it to the expanded CD24^+^ population seen in the embryo.

### The pre-leukemic KMT2A::AFF1 signature is transferable to leukemic patient samples

Finally, to explore the relevance of the identified signature in human disease, the genes were converted to their human counterparts and expression investigated in patient ALL samples. When analyzing bulk RNA-seq data from different subtypes of ALL [46], the pre-leukemic signature was found significantly enriched in KMT2A::AFF1 patients (Fig. 6E). To evaluate expression patterns of the signature in infant KMT2A::AFF1 ALL, two single-cell RNA-seq data sets [47, 48] were investigated. To resolve the hierarchy among the leukemic blasts, cells were first mapped onto a healthy BM reference [48] (Fig. 6F). Signature positive cells were found to variable degree in the patients, but not in the healthy control. Overall, the positive cells primarily projected to Common Lymphoid Progenitor (CLP) and PreProB cell types on the healthy reference, linking it to the murine pre-leukemic population (Fig. 6G–I, Supplementary Fig. 8D–F, Supplementary Table 4). The frequency of positive cells however differed between the datasets investigated, which could be due to differences in purification or methodology. We also detected the signature in some patients with KMT2A::MLLT1 or KMT2A::MLLT3 rearrangements (Fig. 6J; Supplementary Table 4), suggesting that the signature can be broadly applicable to KMT2A rearranged leukemia.

*Hmga2* is, as mentioned, associated with fetal hematopoiesis [41–44] and a potential target of KMT2A::AFF1 [26, 45], making it an essential part of the pre-leukemic signature. We therefore explored expression of *HMGA2* in different sub-types of ALL [46], and found it upregulated in KMT2A::AFF1-driven leukemia and enriched in infants. *HMGA2* also played an important role in expansion of leukemic cells as its knock down in KMT2A::AFF1 cell lines reduced proliferation, consistent with a previous study [45] (Fig. 6K**;** Supplementary Fig. 8G). As the murine pre-leukemic PreProB cells could be defined by CD24^+^CD19^-^ and expression of *Hmga2*, we explored the existence of an equivalent subset in the human setting. In the healthy BM control, *HMGA2* expression was confined to the primitive *CD34* positive HSPC cluster, and *CD24* was mainly seen in the B cell compartment, in line with the expression pattern in human B cell development [49] (Supplementary Fig. 8H). Next, in infant ALLs, transcriptionally *CD24*^*+*^*CD19*^*-*^ cells were selected from the single-cell data sets and *HMGA2* expression investigated. Four KMT2A::AFF1 patients (*n* = 11) had a sub-fraction of variable size that also expressed *HMGA2*, a feature that was rare in the KMT2A::MLLT1 and KMT2A::MLLT3 infants (Fig. 6L; Supplementary Fig. 8I, Supplementary Table 4).

Thus, the ontogeny-specific transcriptional program identified in our murine model is enriched in KMT2A::AFF1 patients, providing critical insights into the transcriptional alterations that drive human disease progression.

## Discussion

KMT2A::AFF1 infant ALL stands out as a disease of its own, with a specific clinical manifestation and unfavorable prognosis [3]. Adding to that, murine models have not been able to fully recapitulate the disease [13]. Here, using a novel *KMT2A::AFF1* murine model we obtained novel insights into the disease initiating steps in the embryo of relevance to human disease. By combining the Tet-op promoter with Cre-LoxP technology, we generated an inducible system that allowed for cell-specific expression within the hematopoietic system. Induction of the onco-fusion in the HSPC compartment revealed a bias towards myeloid output in vitro and a selective negative impact on the cloning capacity of HSCs. The former is interesting from a clinical perspective as KMT2A::AFF1 ALL is known to be able to switch lineage upon relapse [50]. The latter feature may be linked to increased levels of *Gata2*, known to induce quiescence in HSCs [38]. *Gata2* activity was most prominent in the more primitive clusters, highlighting that this effect could be cell type specific, which would be in line with our functional data. Additionally, the finding is not specific to the KMT2A::AFF1 onco-fusion but has also been reported for KMT2A::MLLT1 [25]. The cell cycle status of cancer cells is a crucial factor in therapy response, as current treatments primarily target actively dividing cells.

The impact of *KMT2A::AFF1* on the HSPC compartment was independent of developmental stage as we observed similar outcomes in both embryonic and postnatally induced mice. The expansion of the B compartment was however specific to the embryo. The immunophenotype was consistent with PreProBs (B220^+^CD43^+^CD19^-^), except for surface expression of CD24 (also known as heat stable antigen; HSA), a cell adhesion marker normally upregulated later in the B cell hierarchy, on CD19^+^ ProBs [30, 31]. The pre-malignant potential of the *KMT2A::AFF1*^*+*^ CD24^+^ PreProBs was further explored in vivo. As only ∼500-1300 CD24^+^ PreProBs were transplanted to each recipient, the immune-deficient NSG model was used, thereby allowing the cells to expand without competition. In this setting the cells displayed lineage plasticity and self-renewal potential. Other pathological features, such as long-term engraftment, splenomegaly and a block in differentiation were observed. Thus, our model allowed for identification of a novel pre-malignant state, that we extensively characterized. Notably, we did not observe development of acute leukemia in our transplantation setting, and it is likely that additional factors are needed for transformation, at least in the murine setting. Two micro RNAs, miR-130b and miR-128a, have been identified as important co-drivers for the conversion [37], and inflammatory stimulus [12, 51] may also be important for the progression to fulminant disease.

The pre-leukemic cells were exclusive to induction with Vav-Cre, and were not observed upon targeting more lymphoid progenitors. This suggests that the cell of origin of pre-leukemia is found within the HSPC compartment. One could speculate as to why these committed cells were not susceptible to transformation. One possible explanation could be that the accessibility to chromatin of key target genes of KMT2A::AFF1 was restricted at the progenitor stages. In line with this, *Hoxa9* was only marginally increased in CD24^+^ PreProBs from the *KMT2A::AFF1* Rag1-Cre and Mb1-Cre models. Notably, the pre-leukemic cells were absent in postnatally induced adult mice. In contrast, the myeloid skewing observed within the HSPC compartment occurred regardless of developmental stage, suggesting it is a consequence of oncogenic effects on definitive HSPCs. Meanwhile, the expansion of the B cell compartment was specific to the embryonic context and likely driven by fetal, transient progenitors. In line with that, Vav-Cre is expressed early in hematopoiesis, targeting definitive HSCs but also cells from the intermediate wave, including pre-HSCs and MPPs [20]. A recent publication highlights that most cells populating the FL during gestation are from the non-definitive wave, with little contribution from definitive HSCs [52]. These findings suggest that the pre-leukemic cells may originate early, potentially from pre-HSCs or even an earlier wave of hematopoiesis.

Our transcriptional analysis revealed an unexpected heterogeneity in the PreProB subset expressing the onco-fusion. A subset of the *KMT2A::AFF1*^*+*^ PreProBs clustered together, exhibiting upregulation of KMT2A::AFF1 target genes and stemness genes. This cluster expressed *Cd24a*, linking the molecular profile of a self-renewing early B progenitor to the distinct pre-leukemic population we identified functionally and prospectively through our study. Thus, the unique CD24^+^ PreProB population identified here displayed stem cell properties in vivo, as well as a transcriptional stemness signature, likely representing the initiating step of disease development. Furthermore, the pre-leukemic signature was detected to varying degrees in KMT2A-rearranged patient samples, including blasts aligning with the CLP and PreProB compartments in a normal reference. This observation aligns with the pre-leukemic cells from which the signature was derived. The characteristic of *Cd24a-Hmga2* co-expression in the murine model could to some extent be observed in the KMT2A::AFF1 infants, and is interesting from a therapeutic perspective, where knockdown of *HMGA2* reduced proliferation of KMT2A::AFF1 ALL cell lines [45]. Additionally, CD24 is a surface marker explored as a promising cancer immunotherapy target [53, 54], and has also been linked to leukemia initiating cell (LICs) in KMT2A::AFF1 ALL [55]. Thus, the distinct CD24 expression on the pre-leukemic cells presents an attractive therapeutic opportunity to explore further.

Altogether, our model captures an embryonic, in situ pre-leukemic state driven by *KMT2A::AFF1*, providing unique insight into the earliest stages of leukemogenesis. These findings offer a foundation for future strategies aimed at intercepting infant ALL at its developmental origin.

## Supplementary information

Supplemental figures and methods

## Data Availability

The data generated within this study have been deposited on GEO under GSE251658. The human samples used in the study can be found here: single-cell RNA-seq of healthy BM and KMT2A rearranged samples [48] at the Human Tumor Atlas Network (HTAN) (https://humantumoratlas.org/); single-cell RNA-seq of the 6 KMT2A::AFF1 patient samples [47] are all deposited together in the European Genome-phenome Archive (EGA) under EGAS00001003986 (and available upon approval by the Princess Máxima Data Access Committee); bulk RNA-seq of ALL patient samples [46] https://pecan.stjude.cloud/static/hg19/pan-all/BALL-1988S-HTSeq.zip. The code can be found in a Github repository: https://github.com/sara-palo/calderon_et_al.
