# Supplementary material for: Ontogeny-specific induction of the KMT2A::AFF1-fusion drives development of a distinct CD24 positive pre-leukemic state
Source: Leukemia. 2025 Jul 11;39(9):2099–111. doi: 10.1038/s41375-025-02665-9 (PMC12380613; doi:10.1038/s41375-025-02665-9)
Supplement: Supplementary file 1 — Supplemental figures and methods [file 41375_2025_2665_MOESM1_ESM.pdf]

Supplementary data and methods

Ontogeny-specific induction of the KMT2A::AFF1-fusion drives development of a distinct CD24 positive pre-leukemic state

Ariana S. Calderón[1], Roshanak Ghazanfari[1], Zahra Masoumi[1], Shabnam Kharazi[1], Sara Palo[1], Stefan Lang[1], Kristijonas Žemaitis[2], Mohamed Eldeeb[1], Agatheeswaran Subramaniam[2], Shamit Soneji[1], Ronald W. Stam[3], David Bryder[1]*& Charlotta Böiers[1]*#

[1]Division of Molecular Hematology, Lund Stem Cell Center, Lund University, Lund, Sweden

[2] Division of Molecular Medicine and Gene Therapy, Lund Stem Cell Center, Lund University, Lund, Sweden

[3] Princess Maxima Center for Pediatric Oncology, Utrecht, The Netherlands

# lead contact

* Corresponding author email: david.bryder@med.lu.se and charlotta.boiers@med.lu.se

This PDF file includes:

- Supplementary Figures 1-8
- Supplementary Tables 1-7
- Supplementary Methods
- Supplementary References

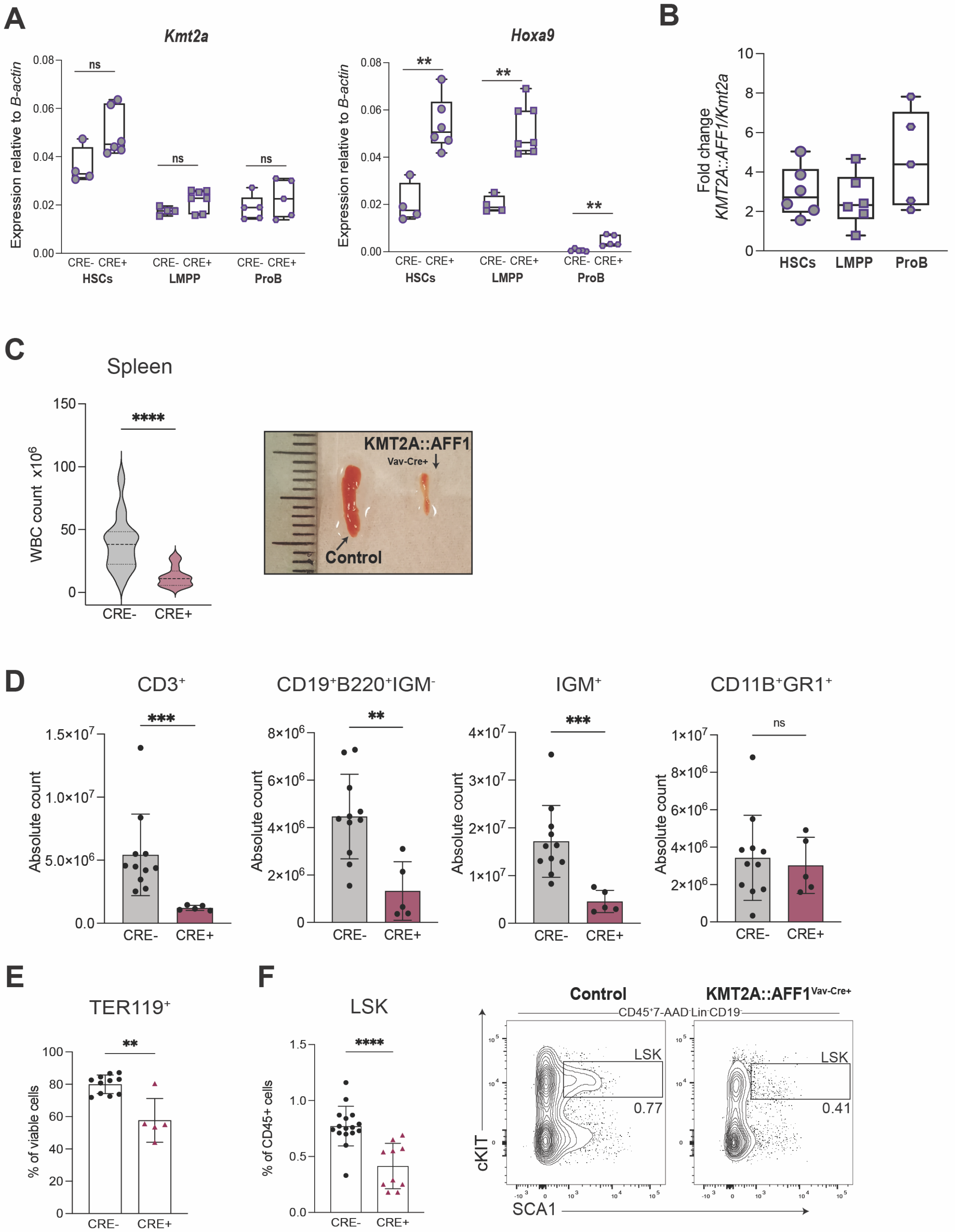

Supplementary Figure 1: Embryonic KMT2A::AFF1 induction affects HSPCs and mature hematopoietic lineages in young pups

(**A**) Relative expression (normalized to *B-actin)* of *Kmt2a and Hoxa9* and (**B**) fold increase in *KMT2A::AFF1* expression compared to endogenous *Kmt2a* in purified HSCs, LMPPs and ProBs from E18.5 *KMT2A::AFF1*$^{\text{Vav-Cre+}}$ fetal livers (FLs). Box plots define lower and upper quartiles, and whiskers min to max values. Each dot represents an individual embryo (4 FACS

experiments). (**C**) Violin plot of spleen white blood cell (WBC) counts from control and *KMT2A::AFF1*$^{\text{Vav-Cre+}}$ pups 3-7 days old (Cre$^{-}_{n=28}$ and Cre$^{+}_{n=13,}$)(7 experiments) (*left*) and representative photos approx. 2 days after birth (*right*). (**D**) Numbers of T (CD3^{+}), B (B220^{+}CD19^{+}IGM^{-} or IGM^{+}) and myeloid (CD11B^{+}GR1^{+}) cells in spleens from control and *KMT2A::AFF1*$^{\text{Vav-Cre+}}$ pups 4-6 days old (3 experiments). (**E**) Frequencies of erythroid (CD45^{-} TER119^{+}) cells in spleens from control and *KMT2A::AFF1*$^{\text{Vav-Cre+}}$ pups as percentage of viable cells. (**F**) Frequencies of LSK compartment in BMs from control and *KMT2A::AFF1*$^{\text{Vav-Cre+}}$ pups 1-6 days old as percentage of CD45^{+} cells (*left*), and representative flow cytometry plots (*right*) (4 experiments). Numbers are mean percentages of total CD45^{+} cells. Bars show means ±SD and each dot represents an individual mouse. ** $p \leq 0.01$; ***$p \leq 0.001$; ****$p \leq 0.0001$; n.s., not significant.

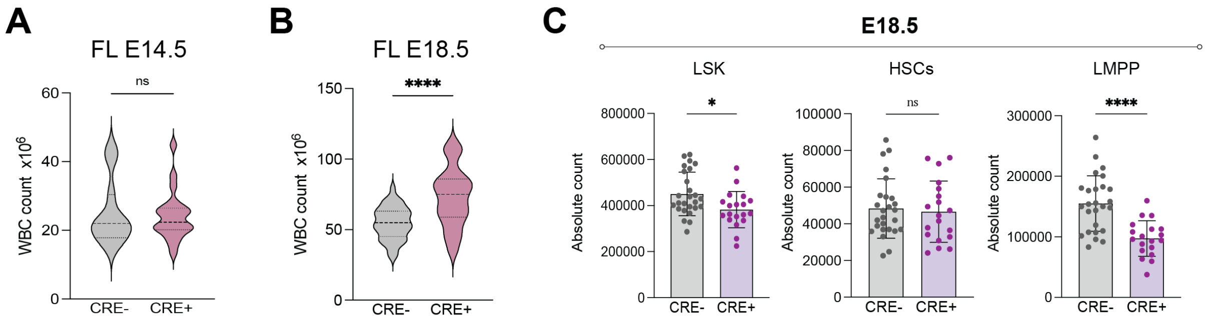

Supplementary Figure 2: HSPC compartment in KMT2A::AFF1$^{\text{Vav-Cre+}}$ embryos

(**A-B**) Violin plots of number of white blood cells (WBCs) in Fetal Livers (FLs) from control and *KMT2A::AFF1*$^{\text{Vav-Cre+}}$ embryos at (**A**) E14.5 (Cre$^{-}_{n=11}$ and Cre$^{+}_{n=21}$) (4 experiments) and (**B**) E18.5(Cre$^{-}_{n=37}$ and Cre$^{+}_{n=35}$)(10 experiments) (**C**) Number of LSK, HSCs and LMPPs in FLs from control and *KMT2A::AFF1*$^{\text{Vav-Cre+}}$ E18.5 embryos (6 experiments). Bars show means ±SD and each dot represents an individual embryo. *p≤0.05; ****p≤0.0001; n.s., not significant.

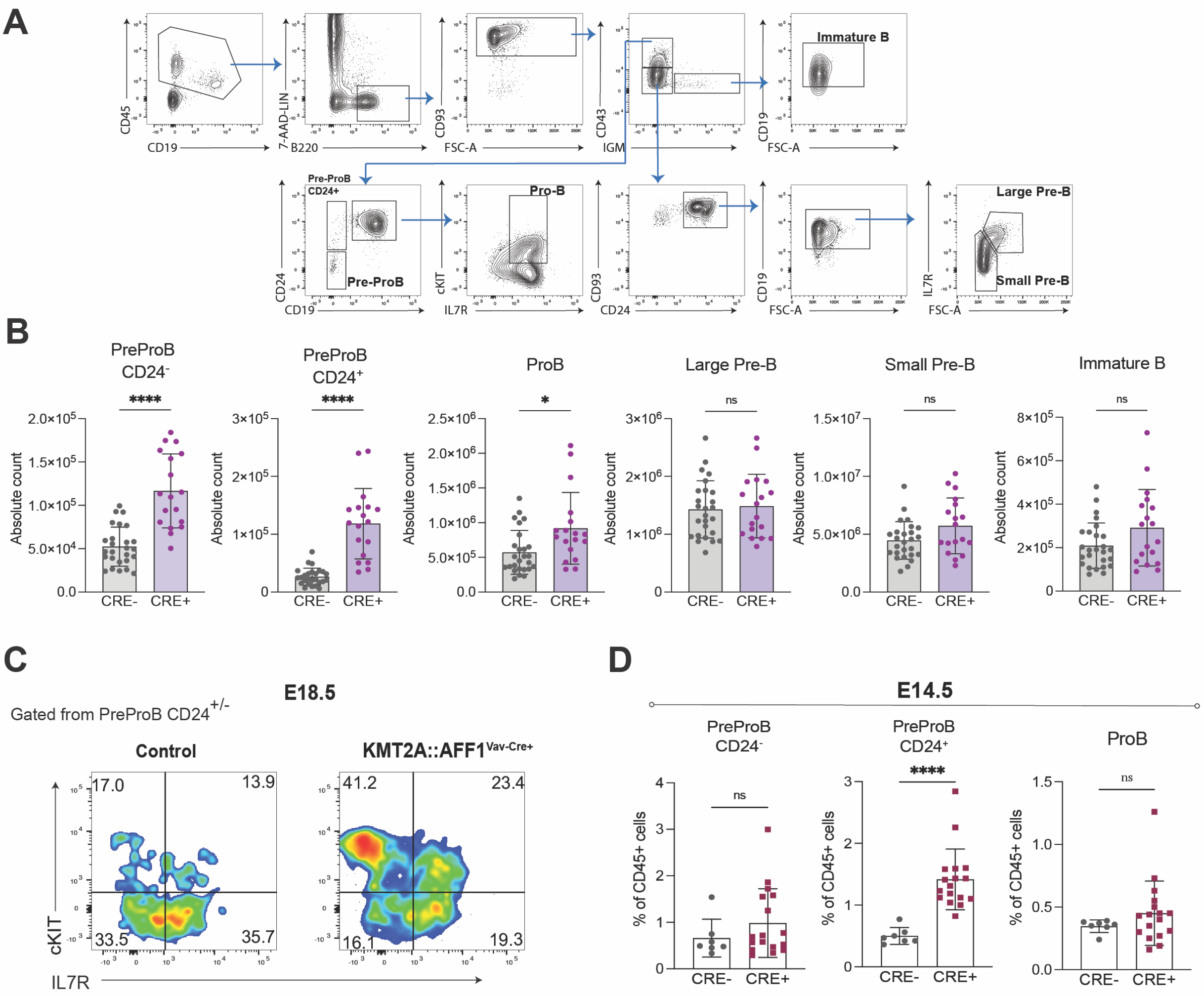

Supplementary Figure 3: B progenitor compartment in KMT2A::AFF1$^{\text{Vav-Cre+}}$ embryos

(**A**) Gating strategy for the fetal B progenitor compartment. FL cells at E18.5 were gated for singlets, size and CD45^{+} cells were selected as shown. Further gating is indicated in the figure and the different B progenitors highlighted. (**B**) Number of different B progenitors in FLs from control and *KMT2A::AFF1*$^{\text{Vav-Cre+}}$ E18.5 embryos (6 experiments). (**C**) cKIT and IL7R immunophenotype of PreProBs (independent of CD24 expression) from control (*left*) and *KMT2A::AFF1*$^{\text{Vav-Cre+}}$ (*right*) E18.5 embryos. Numbers are percentages of parent gate. (**D**) B progenitor compartment in control and *KMT2A::AFF1*$^{\text{Vav-Cre+}}$ embryos at E14.5 displayed as percentage of CD45^{+} cells (3 experiments). Bars show means ±SD and each dot represents an individual embryo. *p≤0.05; ****p≤0.0001; n.s., not significant.

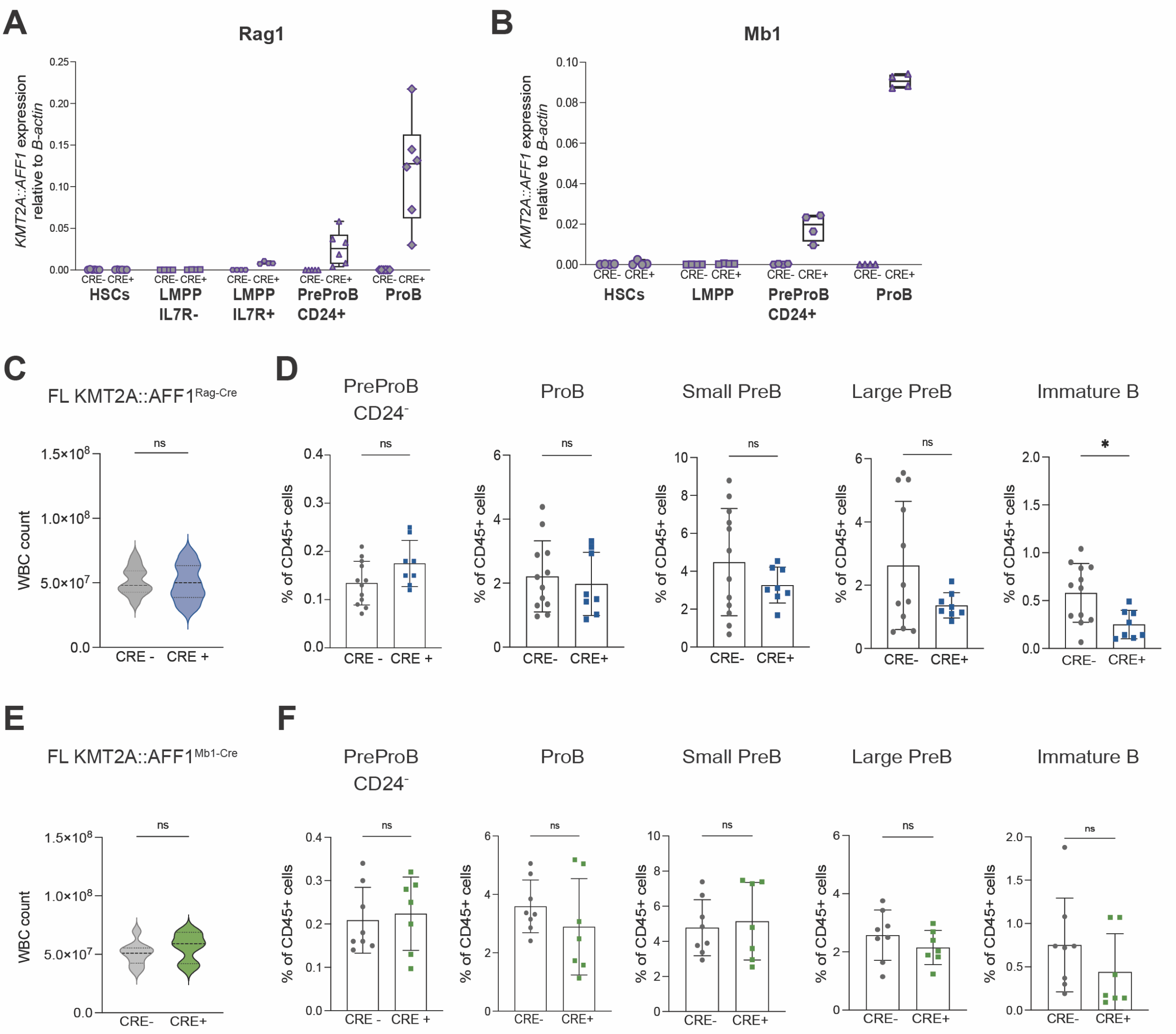

Supplementary Figure 4: B progenitor compartment in Rag1-cre and Mb1-cre KMT2A::AFF1 induced embryos

(**A-B**) Relative expression (normalized to *B-actin)* of *KMT2A::AFF1* in purified HSCs, LMPPs, CD24^{+} PreProBs and ProBs from controls and (**A**) *KMT2A::AFF1*$^{\text{Rag1-Cre+}}$ and (**B**) *KMT2A::AFF1*$^{\text{Mb1-Cre+}}$ at E18.5. Box plots define lower and upper quartiles, and whiskers min to max values. (*KMT2A::AFF1*$^{\text{Rag1-Cre+}}$ embryos were heterozygous or homozygous for *KMT2A::AFF1*$^{\textit{Cola1-tetO}}$) (2 FACS experiments for each strain) (**C**) Violin plot of number of white blood cells (WBCs) in FLs from control and *KMT2A::AFF1*$^{\text{Rag1-Cre+}}$ embryos at E18.5 (Cre$^{-}_{n=21}$ and Cre$^{+}_{n=17}$) (6 experiments). (**D**) Frequencies of B progenitors as percentage of CD45^{+} cells in control and *KMT2A::AFF1*$^{\text{Rag1-Cre+}}$ embryos at E18.5 (4 experiments). (**E**) Violin plot of number of WBCs in FLs from control and *KMT2A::AFF1*$^{\text{Mb1-Cre+}}$ embryos at E18.5(Cre$^{-}_{n=9}$ and Cre$^{+}_{n=7}$) (3 experiments). (**F**) Frequencies of B progenitors as percentage of CD45^{+} cells in control and *KMT2A::AFF1*$^{\text{Mb1-Cre+}}$ embryos at E18.5 (3 experiments). Bars show means ±SD and each dot represents an individual embryo *p≤0.05; n.s., not significant.

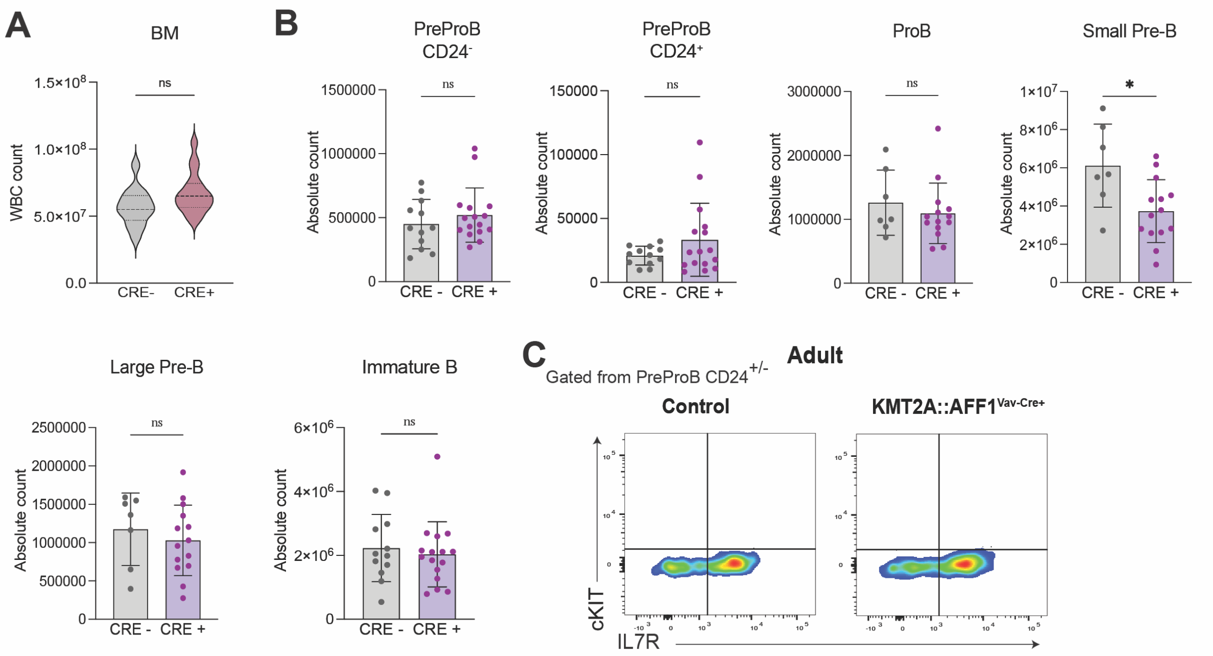

Supplementary Figure 5: B progenitor compartment in postnatally induced adult KMT2A::AFF1$^{Vav\text{-}Cre+}$ mice

(**A**) Violin plot of number of white blood cells (WBCs) in BM (1 tibia+ 1 femur) from control and *KMT2A::AFF1*$^{\text{Vav-Cre+}}$ postnatally induced mice (Cre$^{-}_{n=13}$ and Cre$^{+}_{n=17}$) (8 experiments). (**B**) Number of different B progenitors in BMs from control and *KMT2A::AFF1*$^{\text{Vav-Cre+}}$ postnatally induced mice (6 experiments). (ProBs not defined by cKIT expression). (**C**) cKIT and IL7R immunophenotype of PreProBs (independent of CD24 expression) from control (*left*) and *KMT2A::AFF1*$^{\text{Vav-Cre+}}$ (*right*) postnatally induced mice. Bars show means ±SD and each dot represents an individual mouse. *p≤0.05; n.s., not significant.

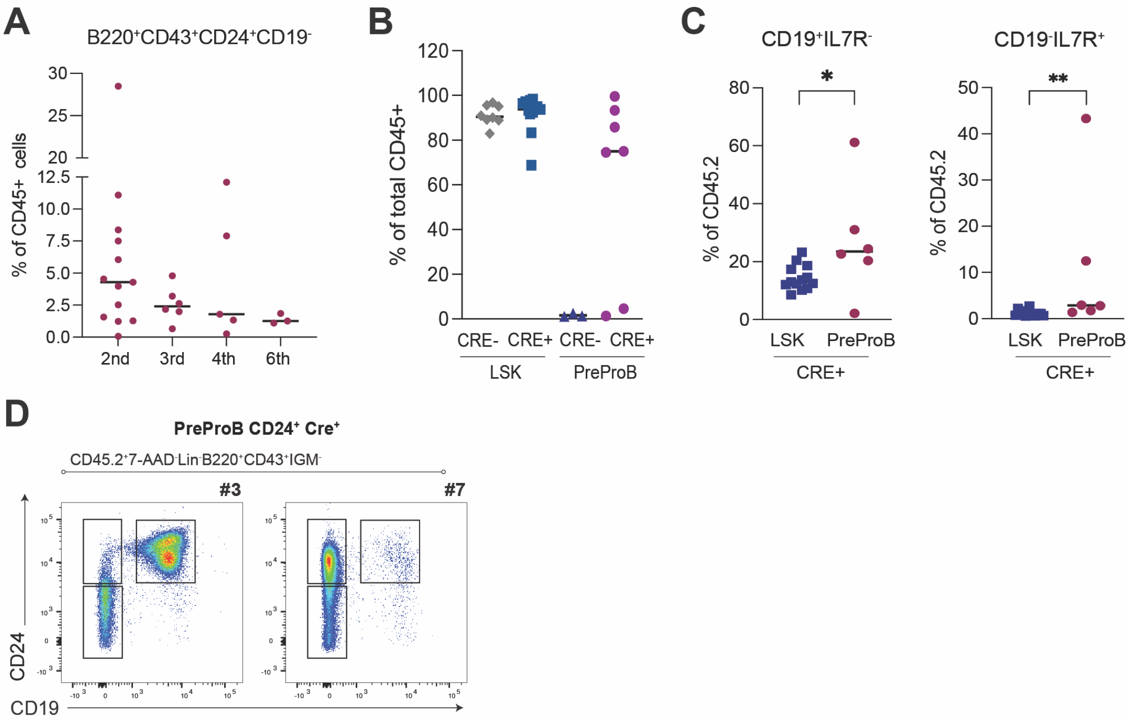

Supplementary Figure 6: Self-renewal potential of KMT2A::AFF1⁺ CD24⁺ PreProBs

(**A**) The immunophenotype of *KMT2A::AFF1*$^{+}$ CD24^{+} PreProBs cultured in methylcellulose was analyzed with flow cytometry at replating. Frequency of cells with a B220^{+}CD43^{+}CD24^{+}CD19^{-} immunophenotype is shown as percentage of total CD45^{+} (4 experiments). (**B**) LSK and CD24^{+} PreProBs from control and *KMT2A::AFF1*$^{\text{Vav-Cre+}}$ were transplanted into sub-lethally irradiated NSG mice. Frequencies of donor cells (CD45.2) in BM of recipient mice analyzed 2-8 months after transplantation, shown as percentage of total CD45. (**C**) Frequencies of donor derived Lin^{-}CD19^{+}IL7R^{-} and Lin^{-}CD19^{-}IL7R^{+} cells in BMs of engrafted recipients transplanted with *KMT2A::AFF1*$^{\text{Vav-Cre+}}$ LSK or CD24^{+} PreProBs shown as percentage of total CD45.2. (**D**) Flow cytometry of B progenitor compartment in two recipients (#3 and #7) transplanted with *KMT2A::AFF1*$^{+}$ CD24^{+} PreProBs. Cells were gated CD45.2^{+}7-AAD^{-}Lin^{-}B220^{+}CD43^{+}IGM^{-}. Lines indicate median and each dot represents an individual embryo/mouse. *$p \leq 0.05$, ** $p \leq 0.01$.

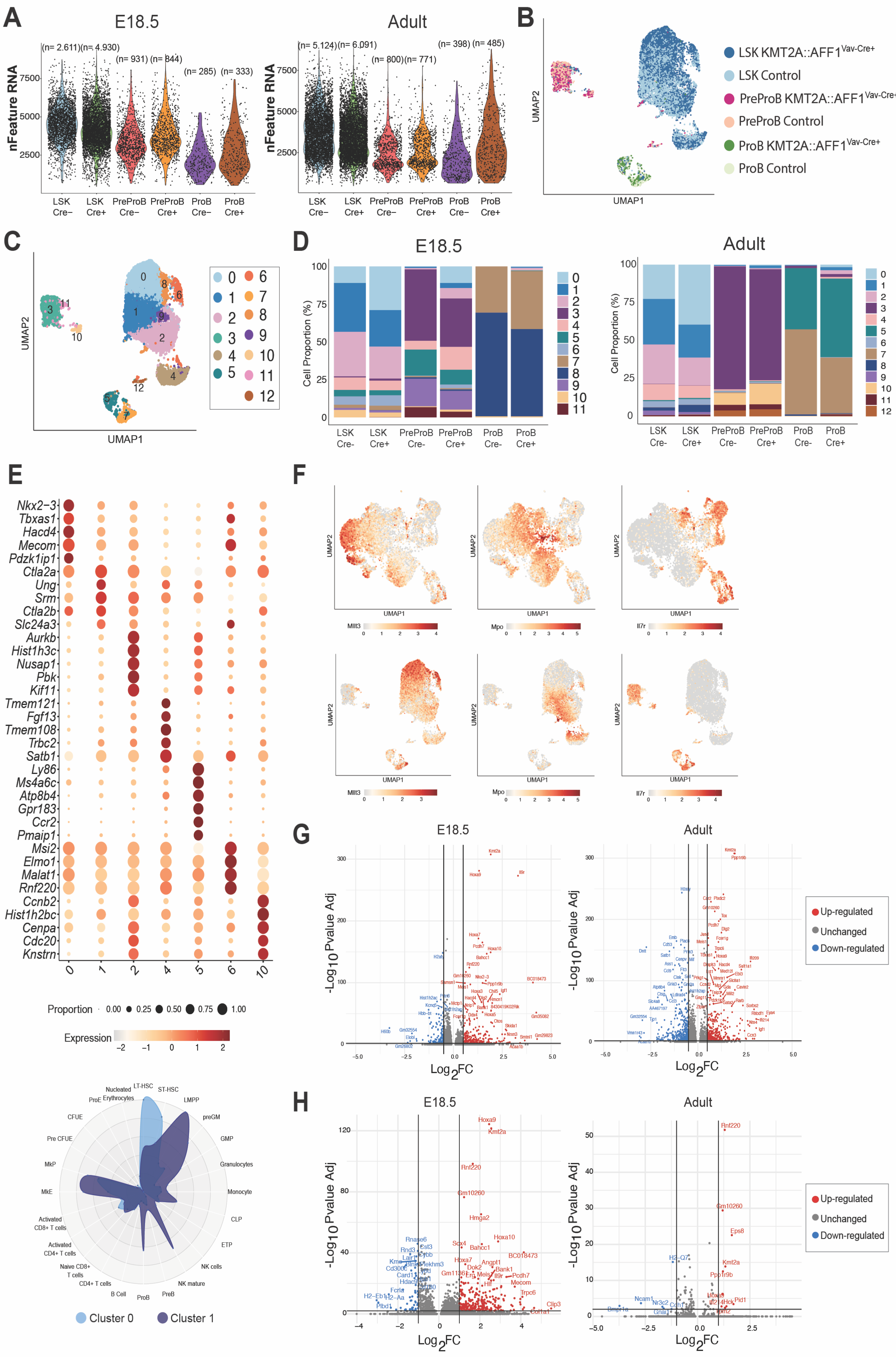
A
E18.5
Adult
nFeature RNA
LSK Cre−
LSK Cre+
PreProB Cre−
PreProB Cre+
ProB Cre−
ProB Cre+
B
UMAP1
UMAP2
LSK KMT2A::AFF1 Vav-Cre+
LSK Control
PreProB KMT2A::AFF1 Vav-Cre+
PreProB Control
ProB KMT2A::AFF1 Vav-Cre+
ProB Control
C
D
Cell Proportion (%)
E
Proportion
Expression
Cluster 0
Cluster 1
F
Mllt3
Mpo
Il7r
G
Log10Pvalue Adj
Log2FC
Up-regulated
Unchanged
Down-regulated
H

Supplementary Figure 7: KMT2A::AFF1 induces transcriptional changes in the HSPC compartment

(**A**) Violin plot of nFeature RNA counts of control and *KMT2A::AFF1*$^{\text{Vav-Cre+}}$ E18.5 (*left*) and postnatally induced adult mice (*right)* for each population sorted. Number of cells analyzed is indicated above each sample. (**B-C**) UMAPs of LSK, PreProBs and ProBs from control and *KMT2A::AFF1*$^{\text{Vav-Cre+}}$ postnatally induced adult mice. (B) Colors coded based on purified population and genotype, and (C) based on cluster. (**D**) Cluster distribution per sorted population and genotype for E18.5 embryos *(left)* and postnatally induced adult mice *(right).* (**E**) Dot plot displaying the top 5 up-regulated genes per cluster in the LSK population for the E18.5 embryos *(top)* and lineage affiliation of top 10 up-regulated genes in cluster 0 and 1, analyzed with CellRadar *(bottom).* (**F**) UMAPs of LSK, PreProB and ProB cells from control and *KMT2A::AFF1*$^{\text{Vav-Cre+}}$ E18.5 embryos *(top)* and postnatally induced adult mice *(bottom),* highlighting *Mllt3 (left) Mpo (middle)* and *Il7r* (*right)* expressing cells. (**G-H**) Volcano plots of differentially expressed genes (DEGs) between control and *KMT2A::AFF1*$^{\text{Vav-Cre+}}$ LSKs (G) and between control and *KMT2A::AFF1*$^{\text{Vav-Cre+}}$ PreProBs (H) at E18.5 (*left*) and postnatally induced adult mice (*right*). Lines indicate |Log2fold| 0.5 (G) and 1.0 (H).

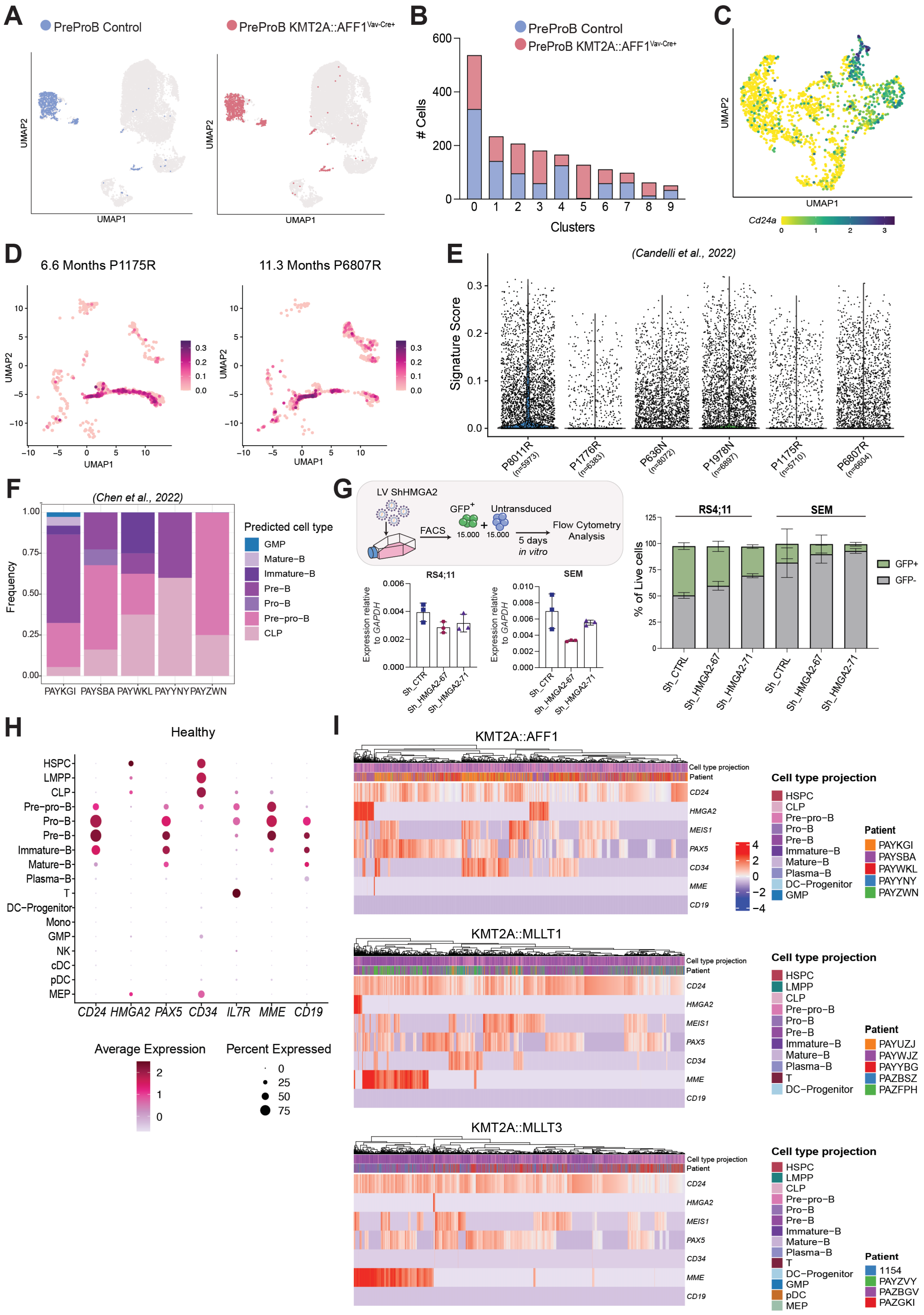
A
PreProB Control
PreProB KMT2A::AFF1Vav-Cre+
B
Cells
Clusters
C
Cd24a
D
6.6 Months P1175R
11.3 Months P6807R
E
(Candelli et al., 2022)
Signature Score
F
(Chen et al., 2022)
Frequency
Predicted cell type
GMP
Mature−B
Immature−B
Pre−B
Pro−B
Pre−pro−B
CLP
G
LV ShHMGA2
FACS
Untransduced
Flow Cytometry Analysis
5 days in vitro
RS4;11
SEM
% of Live cells
GFP+
GFP-
H
Healthy
Average Expression
Percent Expressed
I
KMT2A::AFF1
KMT2A::MLLT1
KMT2A::MLLT3
Cell type projection
Patient

Supplementary Figure 8: The pre-leukemic signature and gene expression in KMT2A::AFF1 leukemic patient samples

(**A**) UMAPs of LSK, PreProBs and ProBs from control and *KMT2A::AFF1*$^{\text{Vav-Cre+}}$ postnatally induced adult mice, highlighting control PreProBs (*left*) and *KMT2A::AFF1*$^{\text{Vav-Cre+}}$ PreProBs (*right*). (**B**) Bar plot displaying cell number per cluster and genotype (control and *KMT2A::AFF1*$^{\text{Vav-Cre+}}$) from the PreProB UMAP in Figure 6B. (**C**) UMAP of PreProBs at E18.5, highlighting *Cd24a* expressing cells. (**D**) Expression of the pre-leukemic signature in individual KMT2A::AFF1 leukemic patient samples from Candelli et al[1], mapped onto the healthy BM UMAP[2] in Figure 6F. Color scale set to the same range for all samples (0.35). Ages of the patients are listed on top. (**E**) The pre-leukemic signature score for individual patients. Number of analyzed cells displayed below each patient. (**F**) Predicted cell type of the signature positive cells in KMT2A::AFF1 leukemic patient samples from Chen et al[2]. (**G**) Schematic illustration of the experiment workflow (*top, left*) *HMGA2* expression in human RS4;11 and SEM ALL cell lines after lentiviral transduction with *HMGA2* shRNAs or control (3 FACS experiments) (*bottom*, *left*). Number of viable GFP positive and negative cells were assessed after 5 days of culture (4 experiments) (*right*) **(H**) Dot plot showing expression of selected genes in the healthy BM control[2]. **(I)** Heatmap showing expression of selected genes in *CD24*$^{+}$*CD19*$^{-}$ cells from KMT2A::AFF1 (*top*) KMT2A::MLLT1 (*middle*) and KMT2A::MLLT3 (*bottom*) leukemic patient samples from Chen et al [2].

SUPPLEMENTARY METHODS

Generation of *Col1a1-tetO-KMT2A::AFF1* Mice

A human *KMT2A::AFF1* oncogene based on GenBank accession L22179.1 was subcloned into the EcoRI site of the pBS31 vector and targeted to KH2 ESCs. Following validation of inducibility of the transgene and karyotyping, the engineered ESCs were injected into E3.5 C57BL/6 blastocysts to generate chimeric mice (Lund University Transgenic Animal Facility). Homozygous *Col1a1-tetO- KMT2A::AFF1* ($KMT2A::AFF1^{Col1a1\text{-}tetO}$) mice were crossed with different Cre strains to be able to generate cell type specific induction of *KMT2A::AFF1* (Vav-Cre[3]; Rag1-Cre[4]; Mb1-Cre[5]). $KMT2A::AFF1^{Col1a1\text{-}tetO}$ were also bred to (*Rosa26-ZtTA*)[6] to generate mice homozygous for tetracycline-trans-activator (tTA) and homozygous for $KMT2A::AFF1^{Col1a1\text{-}tetO}$. Subsequently these mice were bred to generate $KMT2A::AFF1^{Col1a1\text{-}tetO/ZtTA/\text{Strain-Cre+}}$ embryos (named $KMT2A::AFF1^{\text{Strain-Cre+}}$ (Cre^{+})) and $KMT2A::AFF1^{Col1a1\text{-}tetO/ZtTA/\text{Strain-Cre-}}$ littermate controls (named control (Cre^{-})). Cre^{+} embryos were heterozygous for Cre, homozygous for *Col1a1-tetO* and heterozygous for *Zt-TA*, and Cre^{-} embryos were homozygous or heterozygous for *Col1a1-tetO* and heterozygous for *Zt-TA* if not stated otherwise in the text.

Animals

Embryos were obtained by timed mating overnight. The morning after mating was considered E0.5. Postnatal induction of *KMT2A::AFF1* was achieve by feeding doxycycline-containing diet (Doxycycline Food 2g/kg; Ssniff Spezialdiäten) to pregnant females from approx. E5 to E18. Adult mice were analyzed 8-30 weeks of age if not otherwise stated. $KMT2A::AFF1^{\text{Vav-Cre+}}$ males were fragile for unknown reasons and were all analyzed at 8-11 weeks (n=4), whereas females were 13-30 weeks (n=10-12). All animal experiments were performed in accordance with the relevant guidelines and regulations and were approved by the Malmö/Lund Ethics Committee on Animal Testing at the Lund District Court.

Genotyping

DNA was extracted from ear punches using the Extracta™ DNA prep kit. Genomic DNA was amplified using the primers listed in the **Supplementary Table 5** together with the AccuStart II GelTrack PCR SuperMix. PCR products were electrophoresed on 2% agarose gels and

visualized using MIDORI Green Advance together with a DNA ladder (1kb, Thermo Scientific™).

Single-Cell Preparations

Fetal livers (FLs) were dissected and mechanically dissociated and passed through a 40 µm filter. Bone marrows (BMs) were extracted from hind limbs, hip bones, forelimbs, and spine collected in Dulbecco's Phosphate-Buffered Saline (DPBS) supplemented with 2% fetal bovine serum (FBS) and 2 mM Ethylenediaminetetraacetic acid (EDTA). The bones were crushed using a mortar and pestle and passed through a 40 µm filter to obtain single-cell suspensions. Spleens were dissected and mechanically dissociated and passed through a 40 µm filter. Cellularity was counted in Sysmex KX-21N or XQ-320.

Flow Cytometry analysis

FL and adult BM cells were treated with anti-CD16/32 (Fc) blocking antibodies followed by staining with fluorophore-conjugated antibodies. To analyze HSPCs the following antibodies were used: lineage markers (GR1, TER119, NK1.1, CD3 and CD11B (only adult BM)); SCA-1, cKIT, CD150, CD48, CD135, CD127, CD45, CD43 and CD19. To analyze the B progenitor, compartment the following antibodies were used: lineage markers (GR1, TER119, NK1.1, CD3 and CD11B (only adult BM)); CD19, B220, CD127, CD24, IGM, CD45, cKIT, CD93 and CD43.

Spleen cells were treated with anti-CD16/32 (Fc) blocking antibodies followed by staining with the following antibodies: GR1, CD11B, CD45, IGM, cKIT, CD3, CD43, CD19, B220 and TER119. For Peripheral blood (PB) lysis of red blood cells was performed using Ammonium Chloride Solution (Stem Cell Technologies). The cells were then Fc-blocked and antibodies against TER119, CD19, CD11B, GR1, B220, CD3, CD45.1, CD45.2, CD43 and cKIT added to assess blood cell lineage distribution. Detailed information regarding antibodies used are provided in **Supplementary Table 6.**

In all flow cytometry experiments, cells were incubated with 7-amino-actinomycin D (7-AAD) briefly before analysis to stain dead cells. Through the whole procedure, the staining buffer was kept ice-cold, and the cells were stained for 40 min at 4°C in the dark. All experiments

were performed on LSRFortessa X-20 (BD Biosciences) instruments at Lund Stem Cell Center FACS Core Facility. Data analysis was performed in FlowJo v10 (BD Biosciences).

Cell Sorting

FL and BM cells were depleted using a cocktail of biotinylated lineage antibodies (TER119, CD3 and GR1) followed by incubation with anti-biotin conjugated MicroBeads (Miltenyi Biotec). Magnetically labeled cells with Microbeads were removed using the autoMACS Pro Separator and LS columns, according to manufacturer's instructions (Miltenyi Biotech). Staining was done as described above. Cell sorting was performed on FACSAriaIII (85 um nozzle) (BD Biosciences) at Lund Stem Cell Center FACS Core Facility.

Co-culture assay

In single-cell co-culture experiment fetal and adult HSCs, LMPP and $CD24^{+}$ PreProB cells were sorted as bulk and manually plated (60 cells into 600uL of medium; 10ul plated per well) onto a pre-established layer of OP9 cells (2000 cells per 96 well prepared ~24h prior the experiment). Complete medium contained OPTIMEM supplemented with 1% penicillin/streptomycin, 2-Mercaptoethanol (2-ME) 10^{-4} M, 10% FBS and cytokines; 25 ng/mL murine stem cell factor (mSCF), 25 ng/mL murine fms-related tyrosine kinase 3 ligand (mFLT3L), 25 ng/mL murine interleukin 7 (mIL7), 25 ng/mL murine interleukin 3 (mIL3) (only first week for HSCs and LMPP cultures). Detailed information regarding products used are provided in **Supplementary Table 7**. Cells were maintained in an incubator at 37°C and 5% CO_2. Half media change was performed weekly. Cultures were analyzed after 3 weeks for HSCs, 2 weeks for LMPPs and 10 days for PreProBs. Wells were manually scored and wells with growth harvested, Fc-blocked and stained with the following antibodies: GR1, CD11B, CD45, B220, IGM, CD19, CD43 and analyzed by flow cytometry. Cells were assigned to a lineage based on the following markers: B; $CD45^{+}CD19^{+}B220^{+}$ and myeloid; $CD45^{+}CD19^{-}B220^{-}CD11B^{+}GR1^{+}$.

Liquid cultures

For bulk liquid culture experiments, 10 HSCs and 20 LMPPs from E18.5 FLs were FACS-sorted into 100uL of Iscove's Modified Dulbecco's Medium (IMDM) supplemented with 1% penicillin/streptomycin, 1% GlutaMAX, 10% FBS and manually plated onto a 96-well plate. The following cytokines were added: 25 ng/mL mSCF, 25 ng/mL mFLT3L, 25 ng/mL mIL7,

25 ng/mL mIL3 and 25 ng/mL murine thrombopoietin (mTPO). Cells were maintained in an incubator at 37°C and 5% CO_2, photos were taken with an Olympus CKX53 after 7 days of culture and scale bars added using ImageJ software.

Colony-Forming assays

In vitro semi solid cultures were performed with ~100-250 cells sorted into 120uL of IMDM supplemented with 1% penicillin/streptomycin, 1% GlutaMAX, 20% FBS and 2-Mercaptoethanol (2-ME) 10^{-4} M. All cells were transferred to 1.2 ml M3231 Methocult (STEMCELL Technologies) supplemented with 25 ng/mL mSCF, 25 ng/ mFLT3L, 25 ng/mL mIL7, 25 ng/mL mIL3 and from this, 1 ml was plated into one dish. Colonies were scored and replated weekly (typically with around 800 cells/dish). Means were calculated when more than one dish was evaluated per donor. At certain time-points cells were analyzed with flow cytometry. After harvest and Fc -blocking, the cells were stained with GR1, CD11B, CD45, CD24, CD19, CD43, cKIT and B220. Cells were assigned to a lineage based on the following markers: B; $CD45^+CD19^+B220^+$ and myeloid; $CD45^+CD19^-B220^-CD11B^+GR1^+$.

Transplantation assay

Recipient NSG (NOD-SCID-GAMMA ($Il2rg^{-/-}$)) mice (CD45.1) were sub-lethally irradiated (250cGy) at least 4 hours prior to transplantation. Mice received antibiotic prophylaxis (Ciprofloxacin, HEXAL, 125 mg/l in drinking water) for two weeks beginning on the day of irradiation. Donor $CD24^+$ PreProBs cells (~500-1300cells/recipient) or LSK (~1500-2000 cells/ recipient) were FACS purified and subsequently transplanted through intravenous tail vein injection. Cells from one or sometimes two donors were injected per recipients. Total of 8 independent experiments, not all populations were done simultaneously. Experiments where <500 $CD24^+$ PreProB cells were injected/recipient are not included as no reconstitution was seen (*data not shown*).

At different intervals after transplantation peripheral blood was collected from tail vein into EDTA-coated tubes (Sarstedt) to monitor donor-derived reconstitution by flow cytometry as described in '*Flow Cytometry analysis*'. At end-point BMs and spleens were collected and stained as described in '*Flow Cytometry analysis*' with the following changes: BM cells were stained for HSPCs as well as B progenitor compartment with CD45.2 instead of CD45. Lineage markers were GR1, TER119, NK1.1, CD3 and in some experiments also CD11B. Spleen

samples were stained as for the B progenitor compartment with CD45.2 instead of CD45. Reconstitution was assessed by staining with CD45.1 and CD45.2 and viability only. Donor cells were CD45.2 or CD45.2/1. Mice were considered reconstituted when >3% donor cells and >300 total CD45.2^{+} cells were detected. If more than two recipients were transplanted with the cells from the same donor, values are presented as means.

Morphological analysis

Cells of interest were spun onto a glass slide (1000rpm) using a Shandon Cytospin (Thermo Scientific) and air-dried. Slides were stained for 5 min in May-Grünwald solution (Sigma-Aldrich), washed in deionized water, and stained for 20 min in Giemsa stain (Sigma-Aldrich) diluted 1:20 with deionized water. Slides were rinsed in deionized water, air-dried, and visualized with an Olympus BX51 microscope.

Gene Expression Analysis

Cells were FACS-purified directly into 100uL lysis buffer and total RNA was isolated using single-cell RNA purification kit (Norgen Biotek) and converted to cDNA using Super Scrip IV VILO (Invitrogen). qRT-PCR reactions were run with EvaGreen (BioRad) on a CFX96 Touch Real-Time PCR Detection System (Bio-Rad) and for human cell lines qRT-PCR reactions were run with TaqMan^{TM} Gene Expression Master Mix (Applied Biosystems) on a QuantStudio1 (Applied Biosystems) using the primers listed in **Supplementary Table 5**. Data are shown as means of technical duplicates. All signals were quantified using the ΔCt method and were normalized to *β-actin* or *GAPDH* mRNA expression levels.

Cell cycle analysis

FL and BM cells were stained with the following antibodies: lineage markers (GR1, TER119, NK1.1, B220, CD3 and CD11B (adults only)) and SCA-1, CD150, CD48 and cKIT followed by Zombie-Red staining to stain dead cells. Fixation and permeabilization was done using 1% paraformaldehyde and saponin. Intracellular staining was done using Ki-67 antibody and 4,6-Diamidino-2 Phenylindole Dihydrochloride (DAPI).

Human cell lines and lentiviral transduction

Human RS4;11 (received from another research group) was cultured in Alpha-MEM (Gibco) and human SEM (from DSMZ) was cultured in IMDM (Gibco) both supplemented with 10% FBS + 1% penicillin/streptomycin. All cells were cultured in a humidified, 5%CO2 incubator at 37°C. The cell lines were tested negative for Mycoplasma (EZ-PCR^{TM} Mycoplasma Detection Kit).

Short hairpin RNAs (shRNAs) targeting *HMGA2* (HMGA2 shRNA67[7] and 71; **Supplementary Table 5**) or control (shCTRL[7]) were cloned in a pLKO1-GFP vector from The RNAi Consortium (TRC) and lentiviral vectors produced [8]. For lentiviral transduction 200.000 cells from each cell line were aliquoted and incubated in a 24-well plate with the lentivirus overnight. After 5 days 15.000 GFP^{+} cells were purified for gene expression analysis and for co-culture with 15.000 non-transduced cells for 5 days after which viable (7-AAD^{-}) GFP positive and negative cells were analyzed by flow cytometry. Cultures were run in duplicates and data are presented as means.

Single-cell CITE-seq library generation and sequencing

Cells were lineage depleted as described in section '*Cell Sorting'*. To purify LSK the following antibodies were used: lineage markers (GR1, TER119, NK1.1, and CD3); SCA-1, cKIT, CD45, and CD19. To purify PreProBs and ProBs the following antibodies were used: lineage markers (*as above*), CD19, B220, CD24, IGM, CD45, cKIT, CD93 and CD43. (For immunophenotype of sorted populations see **Supplementary Table 1**). Surface markers were stained together with Antibody-Derived Tags (ADTs) after which cell hashing antibodies (HTO) were added to each tube (**Supplementary Table 6**). Samples were loaded onto the 10x chromium next GEM single cell 3´ V3.1 (10x Genomics) according to the manufacturer's instruction and *Stoeckius et al* to enable CITE-seq[9](cellular indexing of transcriptomes and epitopes). Reverse transcription and cDNA amplification were performed, and the resulting libraries were sequenced on a NOVAseq (Illumina). The BCL files were processed using Cell Ranger mkfastq to yield FASTQ files, which were then processed using Cell Ranger count (v7.0.0) to perform alignment to mm10, as well as filtering, barcode counting and UMI counting. Detailed information regarding samples and sorted cell numbers are provided in **Supplementary Table 1**.

Bioinformatic analysis

Single-cell data was processed using Seurat V5 for R[10]. The ADTs were not used in the analysis. Cells with a mitochondrial read count percentage greater than 10% were removed, and those with overall low read counts were also subtracted (nCount_RNA >1000). The counts were log-normalized, and 1000 highly variable genes (HVGs) were calculated. Principal components were calculated using these 1000 HVGs and used to make a UMAP from the first 20 components after Harmony integration [11]. Clusters were identified using Louvain clustering also utilizing the first 20 PCs. Samples were separated based on HTOs. Data were visualized in ShinyCell[12], where plots were also generated. FindMarkers and FindAllMarkers function in Seurat were used to find DEGs, which were visualized using Volcanoplots (p-adj values that were 0, were set to $\sim 10^{-308}$ to allow for visualization on the plot). Venn diagrams were used to find the shared DEGs between different developmental stages (p-adj<0.01; |log2fold|>0.5). Lineage affiliation of these shared genes and DEGs in KMT2A::AFF1^{+} PreProBs compared to control (p-adj<0.01; |log2fold|>1.0) were further assessed in CellRadar (using data from Bloodspot[13]) (available at https://karlssong.github.io/cellradar/).

Generation of the pre-leukemic signature and validation in human patient samples

The pre-leukemic signature was generated by taking the universal DEGs from 1) PreProB cluster #5 compared to control PreProBs and 2) PreProB cluster #5 compared to control cells in cluster #0 (molecular 'HSCs') on the combined UMAP from **Figure 5B** (p-adj<0.05). Venn diagrams of these DEGs, generated 48 shared up- and 8 shared downregulated genes (**Figure 6C; Supplementary Table 3**). *Kmt2a* was among the upregulated common genes but excluded to avoid confusion between wildtype *KMT2A* and fusion gene transcripts.

To evaluate the signature in human ALL samples, BioMart (Ensembl) was used to translate the gene list from mouse to human. The mouse gene (GRCm39) dataset from Ensembl (release 112) was used to convert the gene list to orthologous human genes, followed by a final manual confirmation. The human orthologous gene signature generated 44 up- and 8 down-regulated genes.

The pre-leukemic signature was evaluated in two single-cell RNA-seq datasets from infant KMT2A rearranged ALL[1, 2], using the healthy BM dataset from Chen et al[2] as a reference. The Candelli et al cohort had 6 KMT2A::AFF1 infants (10xGenomics data from P1776R and

P1175R were not part of the original publication). To allow for projection of the samples from Candelli et al[1] onto the healthy BM reference, a new reference UMAP was generated using dimensions 1:20 of the preexisting PCA. Seurat objects were created for the samples and filtered to remove low quality cells. The samples were then mapped onto the reference using Seurat's FindTransferAnchors and MapQuery functions, using dimensions 1:20 of the PCA.

The Chen et al[2] dataset was subsetted to contain only cells annotated as blasts or progenitors and the preexisting cell type labels from the authors' label transfer were used. In both data sets a score for the pre-leukemic signature was calculated for each cell using the AddModuleScore_UCell function (maxRank = 1500) from the UCell package[14]. For heatmaps, gene expression values were scaled using Seurat's ScaleData function prior to sub-setting for *CD24*$^{+}$*CD19*$^{-}$ cells (cutoff: *CD24*$^{+}$>0.5; *CD19*$^{-}$=0; *HMGA2*$^{+}$>0.3; *HMGA2*$^{-}$$\leq$ 0.3) and KMT2A fusion partners of interest (5 KMT2A::AFF1, 4 KMT2A::MLLT3, 5 KMT2A::MLLT1) in order to retain information about global differences in gene expression (**Supplementary Table 4**). Heatmaps were produced using the ComplexHeatmap package[15].

Bulk RNA-seq data from ALL patients[16] were analyzed using DESeq2[17] (n=67-254 per subtype, age range 0-78 years old). Fusion genes and subtypes of interest were selected and filtered to remove patients of unknown age, before running DESeq and variance stabilizing transformation (vst function). Batch effects due to different sequencing methods were removed using the removeBatchEffect function from the Limma package[18]. The upregulated genes in the signature were converted to hg37 Ensembl IDs using the biomaRt package (43 of the genes were found), and statistical analysis of the mean z-scored expression of the genes was conducted using the ANOVA function in R (aov), followed by the TukeyHSD function to generate the adjusted P values.

Data Analysis

Flow cytometry data were analyzed using FlowJo v10 (BD Biosciences). Differences between two groups were assessed with a two-sided nonparametric Mann-Whitney U test using GraphPad Prism v10. Data are presented as mean ± SD if not otherwise stated. Violin plots display median (dash line) and quartiles (dotted line). Experiments were performed without randomization or blinding and no pre-estimation of sample size was performed. Number of independent biological replicates and experiments are provided in the corresponding figure legends.

Supplementary Table 1: *Samples included in the CITE-seq experiment.*

Sample id	Phenotype	Age	Sex	# donors	# Sorted cells	# Cells analyzed
LSK^{Cre-}	$Lin^{-}SCA1^{+}KIT^{+}$	E18.5	1F / 1M	2	8863	2611
LSK^{Cre+}	$Lin^{-}SCA1^{+}KIT^{+}$	E18.5	2F / 2M	4	16000	4930
LSK^{Cre-}	$Lin^{-}SCA1^{+}KIT^{+}$	7- 9 months	F	3	16045/6949	5124
LSK^{Cre+}	$Lin^{-}SCA1^{+}KIT^{+}$	7- 9 months	F	3	16000/8500	6091
Pre-$ProB^{Cre-}$	$Lin^{-}B220^{+}CD43^{+}CD93^{+}CD19^{-}$	E18.5	1F / 1M	2	3500	931
Pre-$ProB^{Cre+}$	$Lin^{-}B220^{+}CD43^{+}CD93^{+}CD19^{-}$	E18.5	2F / 2M	4	3500	844
Pre-$ProB^{Cre-}$	$Lin^{-}B220^{+}CD43^{+}CD93^{+}CD19^{-}$	7- 9 months	F	3	3500/1800	800
Pre-$ProB^{Cre+}$	$Lin^{-}B220^{+}CD43^{+}CD93^{+}CD19^{-}$	7- 9 months	F	3	3500/1800	771
$ProB^{Cre-}$	$Lin^{-}B220^{+}CD43^{+}CD93^{+}CD19^{+}CD24^{+}$	E18.5	1F / 1M	2	3500	285
$ProB^{Cre+}$	$Lin^{-}B220^{+}CD43^{+}CD93^{+}CD19^{+}CD24^{+}$	E18.5	2F / 2M	4	3500	333
$ProB^{Cre-}$	$Lin^{-}B220^{+}CD43^{+}CD93^{+}CD19^{+}CD24^{+}$	7- 9 months	F	3	3500/1800	398
$ProB^{Cre+}$	$Lin^{-}B220^{+}CD43^{+}CD93^{+}CD19^{+}CD24^{+}$	7- 9 months	F	3	3500/1800	485

Supplementary Table 2*: Shared up- and down regulated genes in KMT2A::AFF1^{+} LSKs compared to control, at E18.5 and adult developmental states.*

**Up-regulated**				
2900052L18Rik	*Fcer1g*	*Hoxa10*	*Myo1d*	*Rhag*
6030498E09Rik	*Gata2*	*Hoxa3*	*Myom1*	*Rhbdf1*
8430419K02Rik	*Gm10260*	*Hoxa9*	*Nkx2-3*	*Rhoc*
Abcg3	*Gm10503*	*Igf1*	*Otos*	*Slc29a3*
Acyp2	*Gm11361*	*Il9r*	*P2rx1*	*Smtnl1*
Ahr	*Gm11772*	*Inpp4b*	*Pcdh7*	*Sorbs2*
Bahcc1	*Gm16140*	*Irak3*	*Pdgfc*	*Tbxas1*
BC051537	*Gm16141*	*Kmt2a*	*Pdgfd*	*Tek*
Bgn	*Gm35082*	*Ksr2*	*Pdzk1ip1*	*Thsd4*
C030037D09Rik	*Gm7030*	*Lhcgr*	*Plcb1*	*Tle2*
C230037L18Rik	*Gng11*	*Maged2*	*Plcl1*	*Tle6*
C530008M17Rik	*Gp9*	*Mamdc2*	*Plekhg1*	*Tmtc2*
Chil5	*Gsn*	*Med12l*	*Plxdc2*	*Treml1*
Dlg2	*Gucy1a1*	*Meis1*	*Ppp1r9b*	*Trpc6*
Emcn	*Gucy1b1*	*Mill2*	*Prkcb*	*Uba7*
Exph5	*Hacd4*	*Mmp16*	*Prkg1*	*Vwf*
Eya2	*Hmcn1*	*Mpg*	*Rcan2*	*Xlr4a*
Fam110c	*Hnf4a*	*Mylk*	*Reps2*	
**Down-regulated**				
1700003C15Rik	*Bok*	*Gm47071*	*Il1r1*	*Sdc1*
1810073O08Rik	*C230035I16Rik*	*Gm49980*	*Kcng1*	*Sell*
4933406J09Rik	*Camk2b*	*Gpc3*	*Lonrf3*	*Sema3d*
Ak4	*Car12*	*Gprin3*	*Lrr1*	*Sgsm1*
Arhgdig	*Ccl3*	*H2-Ab1*	*Maoa*	*Shisa8*
Arpp21	*Ccl9*	*H2afy*	*Mc5r*	*Slc16a11*
Atf3	*Dglucy*	*Hist1h2ac*	*Morrbid*	*Sstr2*
Atg9b	*Emb*	*Hist1h2ap*	*Mpo*	*Tnf*
Aunip	*Gm32554*	*Hist1h2bc*	*Mrvi1*	*Tnfaip1*
Bcl6	*Gm42047*	*Hist1h2bg*	*Prtn3*	*Zfp951*
Bhlhe40	*Gm45479*	*Hist1h4i*	*Pvt1*	

Supplementary Table 3: *The pre-leukemic signature: universal up- and downregulated genes in cluster #5 (related to Figure 6C).*

**Up-regulated**		
8430419K02Rik	*Hoxa10*	*Nkx2-3*
Arid2	*Hoxa5*	*Nrip1*
Bahcc1	*Hoxa7*	*Pcdh7*
Bank1	*Hoxa9*	*Pde3b*
BC018473	*Hspa4*	*Phtf2*
Camk2d	*Igf1*	*Plcb4*
Cdk17	*Il9r*	*Ppp1r9b*
Csnk1g3	*Kansl1l*	*Prkg1*
Diaph3	*(Kmt2a)*	*Ranbp9*
Dlg2	*Macrod2*	*Rnf220*
Elf1	*Magi1*	*Rsbn1l*
Foxp1	*Man1a*	*Slit2*
Gem	*Mctp1*	*Sox4*
Gm35082	*Mecom*	*Thsd4*
Hmcn1	*Meis1*	*Tox*
Hmga2	*Mtmr3*	*Utrn*
**Down-regulated**		
Atp6v0c	*Fau*	*Ppia*
Bmyc	*H2afy*	*Rtraf*
Cox4i1	*Ndufb10*	

Supplementary Table 4*: Single-cell RNA-seq of infant KMT2A rearranged patient samples included from Candelli et al[1] and Chen et al[2]. Number of cells expressing the pre-leukemic signature (signature pos. score > 0.03 and neg. ≤ 0.03) and number of HMGA2 expressing cells in the $CD24^{+}CD19^{-}$ subset (cutoff: $CD24^{+}>0.5$, $CD19^{-}=0$; $HMGA2^{+}>0.3$; $HMGA2^{-}\leq 0.3$).*

**Fusion**	**Patient ID**	**Preleukemic signature**		***$CD24^{+}CD19^{-}$***		**Reference**
		**Neg**	**Pos**	***HMGA2* neg**	***HMGA2* pos**	
KMT2A::AFF1	P1175R	5177	533	385	39	1
	P1776R	6098	285	365	35	1
	P1978N	5267	1630	596	374	1
	P636N	6817	1255	121	2	1
	P6807R	5363	1241	231	1	1
	P8011R	4200	1773	1347	1018	1
	PAYKGI	6880	37	824	86	2
	PAYSBA	3614	31	416	182	2
	PAYWKL	7114	8	624	2	2
	PAYYNY	931	5	23	1	2
	PAYZWN	3080	8	153	4	2
KMT2A::MLLT3	1154	5056	11	2130	10	2
	PAYZVY	140	3	64	0	2
	PAZBGV	4220	169	2041	0	2
	PAZGKI	5761	46	2180	26	2
KMT2A::MLLT1	PAYUZJ	7551	92	535	16	2
	PAYWJZ	6514	25	3523	175	2
	PAYYBG	1367	23	294	0	2
	PAZBSZ	7427	14	1435	22	2
	PAZFPH	6478	218	2378	0	2

Supplementary Table 5: *Oligonucleotides for genotyping, gene expression analysis and shRNA sequences .*

**Oligonucleotides**		**Source**
Cre Rev	5´AGCGTTTTCGTTCTGCCAAT	Integrated DNA technologies
Cre Fw	5´ACGAGTGATGAGGTTCGCAA	Integrated DNA technologies
Rosa 4	5´TCAATGGGCGGGGGTCGTT	Integrated DNA technologies
Rosa 10	5´CTCTGCTGCCTCCTGGCTTC	Integrated DNA technologies
Rosa 11	5´CGAGGCGGATCACAAGCAATA	Integrated DNA technologies
Mb1-Cre Fw	5´CCCTGTGGATGCCACCTC	Integrated DNA technologies
Mb1-Cre Rev	5´GTCCTGGCATCTGTCAGAG	Integrated DNA technologies
SApA Rev	5´GGACAGGATAAGTATGACATCATCAA	Integrated DNA technologies
Col2 Rev	5´AGTCTTGGATACTCCGTGACCATA	Integrated DNA technologies
Col1 Fw	5´TCCCTCACTTCTCATCCAGATATT	Integrated DNA technologies
Kmt2a -F	5´AGCACATGACAATAGGACCA	Integrated DNA technologies
Kmt2a -R	5´ACGGAGGACTGAGAATGCA	Integrated DNA technologies
KMT2A::AFF1_ 1	5´GGGAAAGGAAACTTGGATGG	Integrated DNA technologies
KMT2A::AFF1_ 2	5´TGCCAGTAGTGGGCATGTAG	Integrated DNA technologies
Hoxa9 F	5´ACAATGCCGAGAATGAGAGC	Integrated DNA technologies
Hoxa9 R	5´GTTCAGCGTCTGGTGTTTT	Integrated DNA technologies
B-actin 1	5´CTTCTCCAGGGAGGAAGAGG	Integrated DNA technologies
B actin 2	5´CCACAGCTGAGAGGGAAATC	Integrated DNA technologies
**TaqMan Probe ID**		
HMGA2	Hs00971724_m1	Thermo Fisher
GAPDH	Hs99999905_m1	Thermo Fisher
**shRNA Sequences**		**TRC Number**
Control shRNA shCTRL	CAACAAGATGAAGAGCACCAA	-
HMGA2 shRNA-67	AGGAGGAAACTGAAGAGACAT	TRCN0000021967
HMGA2 shRNA-71	AGTCCCTCTAAAGCAGCTCAA	TRCN0000342671

Supplementary Table 6: *Flow cytometry and hashtag antibodies.*

Reagent	Source	Identifier
**Flow cytometry antibodies**		
TER119- Biotin<br>*Lineage cocktail (Biotinylated)*	Biolegend	Cat#116204, RRID: AB_313705
CD3- Biotin<br>*Lineage cocktail (Biotinylated)*	Biolegend	Cat#100304, RRID: AB_312669
GR-1- Biotin<br>*Lineage cocktail (Biotinylated)*	Biolegend	Cat#108404, RRID: AB_313369
TER119-PE-Cy5<br>*Lineage cocktail*	Biolegend	Cat# 116210, RRID: AB_313711
CD3e-PE-Cy5<br>*Lineage cocktail*	BD Biosciences	Cat# 553065, RRID: AB_394598
GR-1-PE-Cy5<br>*Lineage cocktail*	Biolegend	Cat# 108410, RRID: AB_313375
NK1.1-PE-Cy5<br>*Lineage cocktail*	Biolegend	Cat# 108716, RRID: AB_493590
CD11B-PECy5<br>*Lineage cocktail*	Biolegend	Cat# 101209, RRID: AB_312792
CD41-FITC	Biolegend	Cat# 133904, RRID: AB_2129746
IL7R-PE	Biolegend	Cat# 135010, RRID: AB_1937251
SCA-1-PE-Cy7	BD Biosciences	Cat# 558162, RRID: AB_647253
CD48-APC	Biolegend	Cat#103412, RRID: AB_571997
CD45-A700	Biolegend	Cat# 103128, RRID: AB_493715
c-KIT-A780	eBioscience	Cat#47-1171-82, RRID: AB_1272177
CD135-BV421	BD Biosciences	Cat#562898, RRID: AB_2737876
CD150-BV605	Biolegend	Cat#115927, RRID: AB_11204248
CD19-BV786	BD Biosciences	Cat# 563333, RRID: AB_2738141
B220-FITC	BD Biosciences	Cat# 553087, RRID: AB_394618
CD24-PE-Cy7	BD Biosciences	Cat# 560536, RRID: AB_1727452
IGM-APC	BD Biosciences	Cat# 550676, RRID: AB_398464
CD93-BV421	BD Biosciences	Cat# 747716, RRID: AB_2872195
CD43-BV605	BD Biosciences	Cat# 747726, RRID: AB_2872201
SCA-1-FITC	BD Biosciences	Cat# 553335, RRID: AB_394791
CD150-PE-Cy7	Biolegend	Cat# 115914, RRID: AB_439797
GR-1-FITC	BD Biosciences	Cat# 553127, RRID: AB_394643
CD11B-PE	BD Biosciences	Cat# 553311, RRID: AB_396680
B220-PE-Cy7	BD Biosciences	Cat# 561102, RRID: AB_394335
CD3e-BV421	BD Biosciences	Cat# 562600, RRID: AB_11153670

Ki67-FITC	BD Biosciences	Cat# 556026, RRID: AB_396302
SCA-1-PE	BD Biosciences	Cat# 561076, RRID: AB_394792
CD11B-APC	BD Biosciences	Cat# 553312, RRID: AB_398535
CD19-BUV395	BD Biosciences	Cat# 563557, RRID: AB_2722495
c-KIT-BV786	BD Biosciences	Cat# 564012, RRID: AB_2732005
CD45.1-PE	eBioscience	Cat#12-0453-83, RRID: AB_465676
CD45.2-BV786	BD Biosciences	Cat# 563686, RRID: AB_2738375
CD45.2-PE-Cy7	BD Biosciences	Cat# 560696, RRID: AB_1727494
CD16/32-purified (Fc Block)	BD Biosciences	Cat# 553142, RRID: AB_394656
CD93-BV711	BD Biosciences	Cat# 740750, RRID: AB_2740418
B220-BV421	BD Biosciences	Cat# 562922, RRID: AB_2737894
Ter119-BV786	BD Biosciences	Cat# 740875, RRID: AB_2740526
CD41-BV711	BD Biosciences	Cat# 740712, RRID: AB_2740395
**Hashtag antibodies**		
TotalSeq™-A0301 – Hashtag antibody	Biolegend	Cat#155801, RRID: AB_2750032
TotalSeq™-A0302– Hashtag antibody	Biolegend	Cat#155803, RRID: AB_2750033
TotalSeq™-A0303– Hashtag antibody	Biolegend	Cat#155805, RRID: AB_2750034
TotalSeq™-A0304– Hashtag antibody	Biolegend	Cat#155807, RRID: AB_2750035
TotalSeq™-A0305– Hashtag antibody	Biolegend	Cat#155809, RRID: AB_2750036
TotalSeq™-A0306– Hashtag antibody	Biolegend	Cat#155811, RRID: AB_2750037

Supplementary Table 7: *List of reagents and products used in this study.*

Product	Company	Cat #
Mouse recombinant Stem Cell Factor (mSCF)	PeproTech	250-03
Mouse recombinant Fms-related tyrosine kinase 3 ligand (mFLT3-ligand)	PeproTech	250-31L
Mouse recombinant Thrombopoietin (mTPO)	PeproTech	315-14
Mouse recombinant Interleukin 7 (mIL-7)	PeproTech	217-17
Mouse recombinant Interleukin 3 (mIL-3)	PeproTech	213-13
Ethylenediaminetetraacetic acid (EDTA) 0.5M	Sigma	E7889
Fetal Bovine Serum (FBS) (Lot# 2316548RP)	Gibco	26140087
2-mercaptoethanol (50mM)	Sigma	6250
Trypan Blue (0.4%)	Gibco	15250061
Giemsa	Sigma	48900-1L
May-Grünwald	Sigma	63590-1L
QuadroMACS separator	Miltenyi Biotec	130-090-976
MACS LS Columns	Miltenyi Biotec	130-042-401
Anti-biotin MicroBeads UltraPure	Miltenyi Biotec	130-105-637
MethoCult M3231	Stemcell Technologies	03231
GlutaMAX (100X) supplement	Gibco	35050038
Opti-MEM I with GlutaMAX	Gibco	51985034
Iscove's Modified Dulbecco's Medium (IMDM)	Gibco	12440053
Alpha-MEM	Gibco	12000014

Paraformaldehyde 16%	Thermo Fisher Scientific	43368.9L
Saponin	Sigma	54521
Zombie Red™ Fixable Viability Kit	Biolegend	423109
Pen/Strepcillin-Streptomycin (10000U/ml)	Gibco	15140122
EvaGreen Supermix	Bio- Rad	1725211
DAPI (4′,6-diamidino-2-phenylindole)	Invitrogen	D1306
Doxycycline Food 2g/kg	Ssniff Spezialdiäten	A153D70623
7-Aminoactinomycin D (7-AAD)	BD Biosciences	559925
Extracta™ DNA prep for PCR - tissue	Quantabio	95091-025
AccuStart™ II GelTrack PCR SuperMix	Quantabio	95136-500
Midori Green Advance	Nippon Genetics	MG04
Dulbecco's Phosphate-Buffered Saline (DPBS)	Gibco	14190169
Ammonium Chloride Solution	STEMCELL Technologies	07850
Microvette® 500 EDTA K3E	Sarstedt	20.1341
EZ-PCR™ Mycoplasma Detection Kit	Biological Industries	20-700-20
Single Cell RNA Purification Kit	Norgen Biotek	51800
SuperScript™ IV VILO™ Master Mix	Invitrogen	11766050
TaqMan™ Gene Expression Master Mix	Applied Biosystems	4369016

SUPPLEMENTARY REFERENCES

1. Candelli T, Schneider P, Garrido Castro P, Jones LA, Bodewes E, Rockx-Brouwer D*, et al.* Identification and characterization of relapse-initiating cells in MLL-rearranged infant ALL by single-cell transcriptomics. *Leukemia* 2022 Jan; **36**(1)**:** 58-67.

2. Chen C, Yu W, Alikarami F, Qiu Q, Chen CH, Flournoy J*, et al.* Single-cell multiomics reveals increased plasticity, resistant populations, and stem-cell-like blasts in KMT2A-rearranged leukemia. *Blood* 2022 Apr 7; **139**(14)**:** 2198-2211.

3. Stadtfeld M, Graf T. Assessing the role of hematopoietic plasticity for endothelial and hepatocyte development by non-invasive lineage tracing. *Development* 2005 Jan; **132**(1)**:** 203-213.

4. McCormack MP, Forster A, Drynan L, Pannell R, Rabbitts TH. The LMO2 T-cell oncogene is activated via chromosomal translocations or retroviral insertion during gene therapy but has no mandatory role in normal T-cell development. *Mol Cell Biol* 2003 Dec; **23**(24)**:** 9003-9013.

5. Hobeika E, Thiemann S, Storch B, Jumaa H, Nielsen PJ, Pelanda R, Reth M. Testing gene function early in the B cell lineage in mb1-cre mice. *Proceedings of the National Academy of Sciences of the United States of America* 2006 Sep 12; **103**(37)**:** 13789-13794.

6. Li L, Tasic B, Micheva KD, Ivanov VM, Spletter ML, Smith SJ, Luo L. Visualizing the distribution of synapses from individual neurons in the mouse brain. *PLoS One* 2010 Jul 9; **5**(7)**:** e11503.

7. Kumar P, Beck D, Galeev R, Thoms JAI, Talkhoncheh MS, de Jong I*, et al.* HMGA2 promotes long-term engraftment and myeloerythroid differentiation of human hematopoietic stem and progenitor cells. *Blood Adv* 2019 Feb 26; **3**(4)**:** 681-691.

8. Zufferey R, Nagy D, Mandel RJ, Naldini L, Trono D. Multiply attenuated lentiviral vector achieves efficient gene delivery in vivo. *Nat Biotechnol* 1997 Sep; **15**(9)**:** 871-875.

9. Stoeckius M, Hafemeister C, Stephenson W, Houck-Loomis B, Chattopadhyay PK, Swerdlow H*, et al.* Simultaneous epitope and transcriptome measurement in single cells. *Nature methods* 2017 Sep; **14**(9)**:** 865-868.

10. Hao Y, Hao S, Andersen-Nissen E, Mauck WM, 3rd, Zheng S, Butler A*, et al.* Integrated analysis of multimodal single-cell data. *Cell* 2021 Jun 24; **184**(13)**:** 3573-3587 e3529.

11. Korsunsky I, Millard N, Fan J, Slowikowski K, Zhang F, Wei K*, et al.* Fast, sensitive and accurate integration of single-cell data with Harmony. *Nature methods* 2019 Dec; **16**(12)**:** 1289-1296.

12. Ouyang JF, Kamaraj US, Cao EY, Rackham OJL. ShinyCell: simple and sharable visualization of single-cell gene expression data. *Bioinformatics* 2021 Oct 11; **37**(19)**:** 3374-3376.

13. Bagger FO, Sasivarevic D, Sohi SH, Laursen LG, Pundhir S, Sonderby CK*, et al.* BloodSpot: a database of gene expression profiles and transcriptional programs for healthy and malignant haematopoiesis. *Nucleic Acids Res* 2016 Jan 4; **44**(D1)**:** D917-924.

14. Andreatta M, Carmona SJ. UCell: Robust and scalable single-cell gene signature scoring. *Comput Struct Biotechnol J* 2021; **19:** 3796-3798.

15. Gu Z, Eils R, Schlesner M. Complex heatmaps reveal patterns and correlations in multidimensional genomic data. *Bioinformatics* 2016 Sep 15; **32**(18)**:** 2847-2849.

16. Gu Z, Churchman ML, Roberts KG, Moore I, Zhou X, Nakitandwe J*, et al.* PAX5-driven subtypes of B-progenitor acute lymphoblastic leukemia. *Nature genetics* 2019 Feb; **51**(2)**:** 296-307.

17. Love MI, Huber W, Anders S. Moderated estimation of fold change and dispersion for RNA-seq data with DESeq2. *Genome Biol* 2014; **15**(12)**:** 550.

18. Ritchie ME, Phipson B, Wu D, Hu Y, Law CW, Shi W, Smyth GK. limma powers differential expression analyses for RNA-sequencing and microarray studies. *Nucleic Acids Res* 2015 Apr 20; **43**(7)**:** e47.
